# Elevated β-cell stress levels promote severe diabetes development in mice with MODY4

**DOI:** 10.1530/JOE-19-0208

**Published:** 2019-11-04

**Authors:** Bernadette M Trojanowski, Heba H Salem, Heike Neubauer, Eric Simon, Martin Wagner, Rajkumar Dorajoo, Bernhard O Boehm, Leticia Labriola, Thomas Wirth, Bernd Baumann

**Affiliations:** 1 Institute of Physiological Chemistry, Ulm University, Ulm, Germany; 2 Faculty of Pharmacy, Cairo University, Cairo, Egypt; 3 Faculty of Pharmacy, King Khalid University, Abha, Saudi Arabia; 4 Boehringer Ingelheim Pharma GmbH & Co. KG, Biberach, Germany; 5 Division of Endocrinology, Diabetes and Metabolism, Ulm University Medical Centre, Ulm University, Ulm, Germany; 6 Genome Institute of Singapore, Agency for Science Technology and Research, Singapore, Singapore; 7 Lee Kong Chiang School of Medicine, Nanyang Technological University, Singapore, Singapore; 8 Imperial College London, London, UK; 9 Department of Biochemistry, University of São Paulo, São Paulo, Brazil

**Keywords:** monogenetic diabetes, MODY4, IKK/NF-κB pathway, ER-stress, islet degeneration, islet regeneration

## Abstract

Maturity-onset diabetes of the young (MODY) is a group of monogenetic forms of diabetes mellitus caused by mutations in genes regulating β-cell development and function. MODY represents a heterogeneous group of non-insulin-dependent diabetes arising in childhood or adult life. Interestingly, clinical heterogeneity in MODY patients like variable disease onset and severity is observed even among individual family members sharing the same mutation, an issue that is not well understood. As high blood glucose levels are a well-known factor promoting β-cell stress and ultimately leading to cell death, we asked whether additional β-cell stress might account for the occurrence of disease heterogeneity in mice carrying a MODY4 mutation. In order to challenge β-cells, we established a MODY4 animal model based on *Pdx1* (pancreatic and duodenal homeobox 1) haploinsufficiency, which allows conditional modulation of cell stress by genetic inhibition of the stress-responsive IKK/NF-κB signalling pathway. While Pdx1^+/−^ mice were found glucose intolerant without progressing to diabetes, additional challenge of β-cell function by IKK/NF-κB inhibition promoted rapid diabetes development showing hyperglycaemia, hypoinsulinemia and loss of β-cell mass. Disease pathogenesis was characterized by deregulation of genes controlling β-cell homeostasis and function. Importantly, restoration of normal IKK/NF-κB signalling reverted the diabetic phenotype including normalization of glycaemia and β-cell mass. Our findings implicate that the avoidance of additional β-cell stress can delay a detrimental disease progression in MODY4 diabetes. Remarkably, an already present diabetic phenotype can be reversed when β-cell stress is normalized.

## Introduction

Maturity-onset diabetes of the young (MODY) is a clinically heterogeneous form of diabetes that is inherited in an autosomal-dominant pattern and in general, affected families have a history of diabetes over several generations (Fajans *et al.* 2001). Diagnosis of MODY has important clinical implications for prognosis and therapy but is often misdiagnosed as type 1 or type 2 diabetes mellitus. The prevalence of MODY was reported as 6.5% of antibody-negative diabetes in childhood, in the Norwegian population (Johansson *et al.* 2017) and 1–5% of total diabetes cases (Heuvel-Borsboom *et al.* 2016). Pedigree analysis of MODY families revealed variable age of diabetes onset, which is usually characterized by fasting hyperglycaemia or glucose intolerance. Furthermore, differences concerning disease severity and therapy response can be observed (Fajans & Bell 2011, Bansal *et al.* 2017, Hattersley & Patel 2017, Owen 2018). Interestingly, phenotypic heterogeneity is also found within MODY families harbouring the same gene mutation suggesting the influence of unknown environmental factors in disease development (Fajans & Bell 2006, Fajans *et al.* 2010). Obesity might be one of the critical factors promoting early diabetes onset in MODY4 mutation carriers (Fajans *et al.* 2010). In this condition β-cells are stressed and forced to compensate the increased insulin demand (Ferrannini *et al.* 2004). Thus, the occurrence of any additional β-cell stress might determine disease onset and clinical progression in MODY patients. Mutations in the *PDX1* gene account for the well-characterized MODY4 subtype (Stoffers *et al.* 1997, Ahlgren *et al.* 1998). PDX1 is a transcription factor critically involved in pancreatic development in men and mice (Jonsson *et al.* 1994). In the adult pancreas, PDX1 is crucial for the regulation of β-cell function and homeostasis (Zhu *et al.* 2017). Specific knock-out of *Pdx1* in β-cells results in diabetes development with aging whereas mice haploinsufficient for Pdx1 (Pdx1^+/−^) reflecting MODY4 cases exhibit impaired glucose tolerance but importantly do not develop diabetes (Ahlgren *et al.* 1998, Brissova *et al.* 2002). Interestingly, defective insulin secretion associated with MODY4 in mice is related to the mitochondrial transcription factor TFAM, whose expression is depending on Pdx1 levels in β-cells (Gauthier *et al.* 2009). Thus, reduced Pdx1 activity may define a prediabetic state that is prone for diabetes development (Dutta *et al.* 1998).

Endoplasmic reticulum (ER) stress is recognized as the Achilles heel of pancreatic β-cells and deregulated ER stress signalling is associated with β-cell failure in both polygenic and monogenic forms of diabetes (Cnop *et al.* 2017, Dorajoo *et al.* 2017). ER stress initiates a complex signalling cascade called unfolded protein response (UPR) that activates multiple signalling pathways, which aim to limit ER stress and regain ER homeostasis (Lee & Ozcan 2014, Cnop *et al.* 2017). The IKK/NF-κB signalling system is one of these ER stress-responsive pathways (Nakajima & Kitamura 2013), and NF-κB target genes are important for resolving ER stress and are able to affect β-cell survival (Chan *et al.* 2012, Nakajima & Kitamura 2013).

So far it is not clear whether the extent of β-cell stress could trigger a mild or severe aetiopathology in MODY4 mutation carriers and whether restoration of normal cell homeostasis could improve an already established diabetic phenotype. For this purpose, we have established and characterized a novel Pdx1-haploinsufficient MODY4 mouse model that allows reversible genetic manipulation of β-cell stress.

## Materials and methods

### Experimental mice

Male mice were housed under specific pathogen-free conditions at the animal facility of the University of Ulm. Pdx1.tTA mice (C57BL/6) and (tetO)_7_.IKK2-DN mice (NMRI) were described previously (Holland *et al.* 2002, Baumann *et al.* 2007). Pdx1.tTA mice represent a knock-in model in which the coding sequence of tetracycline-dependent transactivator (tTA) replaced one allele of the endogenous *Pdx1* gene, thereby rendering this mouse line heterozygous for Pdx1 (Pdx1^+/−^). Control littermates include wild-type and single-transgenic (tetO)_7_.IKK2-DN mice unless otherwise stated. For transgene repression in double-transgenic IKK2-DN^Pdx1^ animals doxycycline (Dox; 1 g/l) was administered in the drinking water of all mice as indicated. Mice with *Nemo* deletion in β-cells (Nemo^ΔPanc^) were described previously (Maier *et al.* 2013) and combined with Pdx1.tTA to generate Pdx1^+/−^/Nemo^ΔPanc^ mice. All experiments were performed in compliance with institutional guidelines (TFZ, Ulm) and German animal protection law and approved by Regierungspräsidium Tübingen (Tübingen, Germany).

### Metabolic studies

Mice were fed with a standard chow diet (Supplementary Table 1, see section on supplementary materials given at the end of this article). Fasted blood glucose was measured after a 14-h fasting-period using One-Touch Ultra glucometer (LifeScan, Mipitas, CA, USA). Glucose tolerance was assessed by i.p. injection of 2 mg/g glucose followed by blood glucose measurement after 30, 60, 90 and 120 min. Insulin was determined in plasma samples as described previously (Salem *et al.* 2014) using the Ultra-Sensitive Mouse Insulin ELISA Kit (Chrystal Chem, Elk Grove Village, IL, USA). Plasma glucagon concentration was evaluated using the Cisbio Serum Glucagon Kit (Cisbio, Codolet, France). For glucose-stimulated insulin secretion, ten islets were pre-incubated in Krebs buffer at 37°C, 5% CO_2_ with 5.6 mM glucose for 30 min. Functionality was assessed by stimulation with 2.8 mM and 26.5 mM for 1 h. The insulin released was determined in the cells’ supernatant using the Cisbio Insulin assay following the ‘sensitive’ protocol as suggested by the manufacturer.

### Protein biochemistry

Pancreata were snap-frozen in liquid nitrogen and pulverized, and proteins were extracted for Western immunoblotting and luciferase activity measurement (Baumann *et al.* 2007). Electrophoretic mobility shift assay was performed as described (Baumann *et al.* 2007) using whole-cell protein extract from isolated islets.

### Histology and immunostaining

For paraffin sections, pancreata were processed as previously described (Lattke *et al.* 2012, Salem *et al.* 2014). Sections were incubated with primary antibodies overnight (Supplementary Table 2). Secondary antibodies were coupled with Alexa Fluor (Invitrogen) for immunofluorescence or with horseradish peroxidase that was developed by 3-amino-9-ethylcarbazole (DakoCytomation) for immune-histochemistry. Fibrosis was assessed using Pikro-Siriusrot solution (Morphisto, Frankfurt, Germany). Immunofluorescent stainings were visualized as before (Lattke *et al.* 2012), and other stainings were analysed on a Leica DM IRB microscope (Leica Microsystems) equipped with ProgRes C14 digital camera (Jenoptik).

### Detection of β-cell death

*In situ* detection of DNA strand breaks was performed using the TUNEL labelling method with the FragEL DNA Fragmentation Detection Kit and colorimetric-TdT enzyme (EMD Millipore) according to the manufacturer’s instructions.

### Islet isolation

Pancreata were perfused *in situ* via the common bile duct with 0.5 mg/mL ice-cold collagenase XI solution (Sigma-Aldrich) and isolated as described previously (Salem *et al.* 2014).

### RNA and microarray analysis

RNA was extracted with miRNeasy kit (Qiagen) and cDNA synthesis was done using the Transcriptor High Fidelity cDNA Synthesis Kit (Roche). Quantitative real-time PCR was performed with the Roche LightCycler 480 (Roche) using gene-specific primers and hydrolysis probes designed by the Roche Universal Probe Library system. Microarray analysis was performed with the Mouse Gene 1.0 ST array (Affymetrix) and evaluated with the ‘Genesifter’ software (Geospiza, Seattle, WA, USA). Pathway analysis was performed using the ‘REACTOME’ software, version 3.5 database release 63 (https://reactome.org/). Deregulated genes overlapping with human T2D loci were identified from previous GWAS studies for T2DM and blood glucose measures (GWAS Catalog, https://www.ebi.ac.uk/gwas/; FUMA v1.3.3) (Watanabe *et al.* 2017).

### Statistical analysis

Values are denoted as mean ± s.e.m. Statistical analysis was performed with the GraphPad Prism software ANOVA test. Results were analysed for Gaussian distribution and passed the normality test. The statistical differences between group means were tested by one- or two-way ANOVA when necessary, followed by Bonferroni *post hoc* test as indicated. A value of *P* < 0.05 was considered statistically significant.

## Results

### Establishment of a MODY4 mouse model allowing conditional genetic regulation of β-cell stress

**Figure 1:**
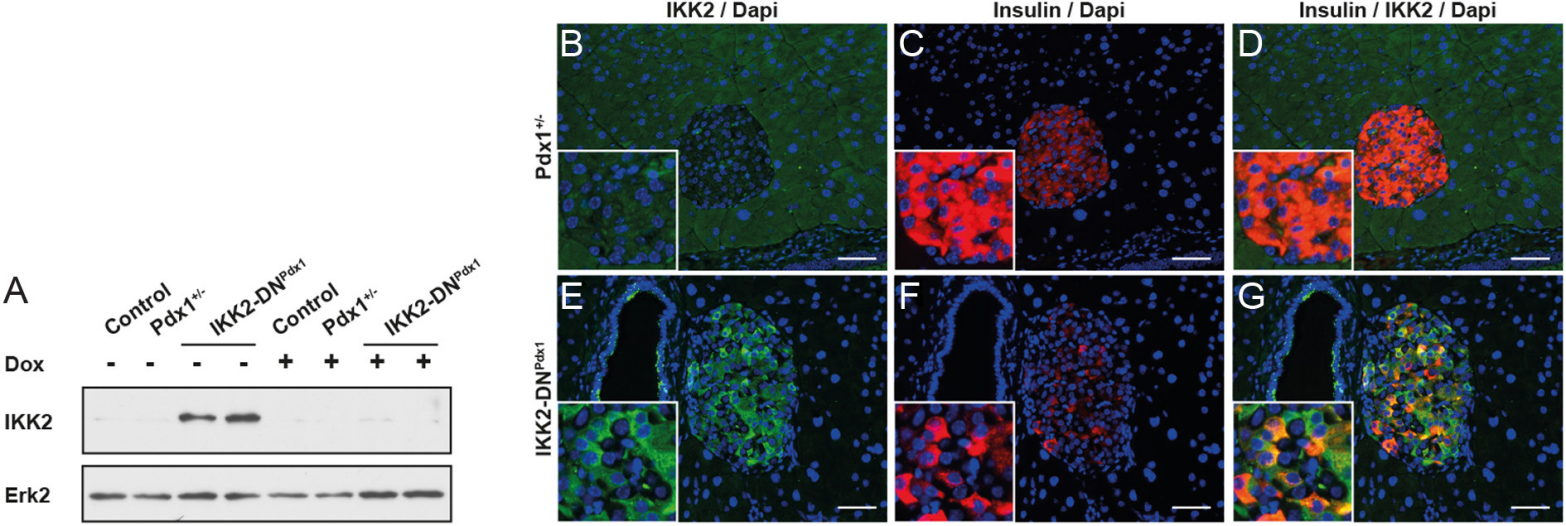
Conditional transgene expression in pancreatic β-cells of mice with Pdx1 haploinsufficiency. Western blot of pancreatic extracts (A) from untreated (−) and DOX-treated (+) mice at the age of 16 weeks. Immunofluorescence staining of pancreatic sections showing expression of the IKK2-DN transgene (green) and insulin (red) (B, C, D, E, F, G, scale bar 50 µm).

To investigate the impact of additional stress factors on disease development in monogenetic forms of diabetes, we created a specific MODY4 mouse model that enables a genetically controlled and reversible induction of β-cell stress. For this purpose we combined the Pdx1.tTA knock-in mouse model (Holland *et al.* 2002), which genocopies MODY4, with luciferase-(tetO)_7_-IKK2-DN mice (Herrmann *et al.* 2005) to generate double-transgenic IKK2-DN^Pdx1^ animals (Supplementary Fig. 1A). This IKK2-DN^Pdx1^ model allows doxycycline-controlled (Dox) regulation of the stress-responsive IKK/NF-κB pathway in pancreatic β-cells together with MODY4 genotype. Accordingly, IKK2-DN transgene was detected only in the pancreas of IKK2-DN^Pdx1^ animals in the absence of Dox but was repressed by Dox application (Fig. 1A). Overall, transgene expression was determined by the measurement of luciferase reporter gene activity revealing strong expression in the pancreas and moderate expression in the intestine (Supplementary Fig. 1B and C). Immunofluorescence staining demonstrated IKK2-DN transgene expression in pancreatic islets (Fig. 1B, C, D, E, F, G and Supplementary Fig. 1E, F, G). As a consequence, NF-κB DNA-binding analysis showed reduced basal and LPS-stimulated NF-κB activity in IKK2-DN^Pdx1^ mice compared to Pdx1^+/−^ littermates (Supplementary Fig. 1D) indicating transgene-dependent inhibition of IKK/NF-κB signalling in islets of these mice.

### IKK/NF-κB inhibition in β-cells of MODY4 mice results in early diabetes development

**Figure 2:**
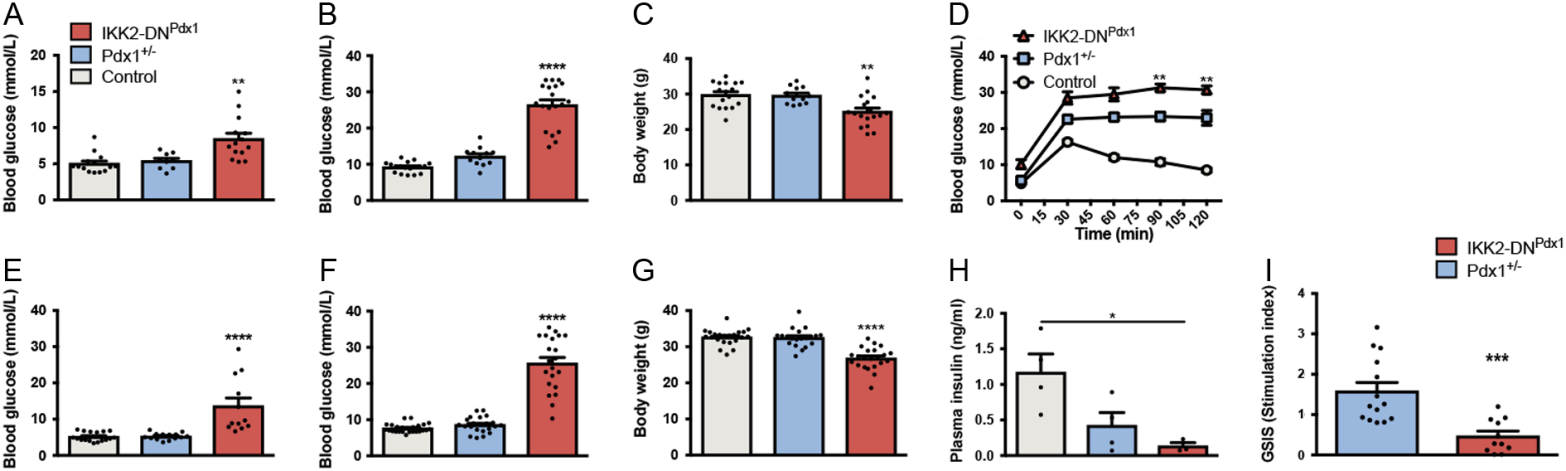
Expression of IKK2-DN in pancreatic β-cells of MODY4 mice results in diabetes development. Fed (A), fasted (B) blood glucose values as well as body weight (C) of 5-week-old control (*n* = 18), Pdx1^+/−^ (*n* = 12) and IKK2-DN^Pdx1^ (*n* = 19) animals. Glucose tolerance test (D) of IKK2-DN^Pdx1^ mice (*n* = 7) compared to Pdx1^+/−^ animals (*n* = 5) at the age of 5 weeks (controls *n* = 6). Fed (E), fasted (F) blood glucose values and body weight (G) of 16-week-old mice (controls *n* = 17, Pdx1^+/−^ *n* = 14, IKK2-DN^Pdx1^ *n* = 12). Plasma insulin levels (H) at the age of 16 weeks (*n* = 4 for control and Pdx1^+/−^ animals, *n* = 3 for IKK2-DN^Pdx1^ mice). Stimulation index (I): ratio between insulin secreted in the presence of high glucose and the release of the hormone during the incubation with the low glucose solution (*n* = 5 independent experiments performed in duplicates or triplicates). Results were analysed by unpaired *t*-test (I), one-way (A, B, C, E, F, G, H, J, K) or two-way (D) ANOVA followed by Bonferroni post-test. Results are presented as the mean ± s.e.m. and compared to Pdx1^+/−^ animals. *P < 0.05; **P < 0.01; ***P < 0.001; ****P < 0.0001.

Consistent with previous findings observed in haploinsufficient Pdx1^+/−^ mice (Ahlgren *et al.* 1998, Brissova *et al.* 2002), Pdx1.tTA knock-in mice were healthy without any changes in body weight and showed an insignificant increase in fed blood glucose levels (Fig. 2A, B, C, E, F and G). However, Pdx1.tTA animals exhibited an impaired glucose tolerance (Fig. 2D) mimicking a mild MODY4 phenotype.

Interestingly, when IKK/NF-κB signalling was inhibited in β-cells, IKK2-DN^Pdx1^ mice developed clear clinical signs of diabetes including polyuria, polydipsia and loss of body weight, detectable already at the age of 5 weeks (Fig. 2A, B, C and Supplementary Fig. 2A). Fasted blood glucose of IKK2-DN^Pdx1^ mice reached diabetic status (8.4 vs 5.4 mmol/L, Fig. 2A) compared to control and Pdx1^+/−^ mice, whereas strong hyperglcaemia (26.4 vs 12.2 mmol/L) was observed under fed conditions (Fig. 2B). Consistent with that, IKK2-DN^Pdx1^ mice showed pronounced glucose intolerance compared to Pdx1^+/−^ mice (Fig. 2D). At 16 weeks fasted blood glucose levels (13.6 vs 5.3 mmol/L) were strongly increased in IKK2-DN^Pdx1^ mice (Fig. 2E and Supplementary Fig. 2A), indicating disease progression. Fed blood glucose (25.6 vs 8.5 mmol/L) remained constantly high (Fig. 2F). At this age Pdx1^+/−^ MODY4 mice had reduced plasma insulin concentration compared to controls (0.42 vs 1.12 ng/mL; Fig. 2H). However, IKK2-DN^Pdx1^ mice displayed a much stronger reduction in plasma insulin (0.13 vs 1.12 ng/mL; Fig. 2H). Additionally, their significant reduction in body weight at all assessed time points further confirmed the prominent diabetic phenotype (Supplementary Fig. 2A). Moreover, a functional impairment in glucose-stimulated insulin secretion (GSIS) was detected in isolated islets from IKK2-DN^Pdx1^ mice compared to Pdx1^+/−^ controls (1.58 vs 0.47; Fig. 2I).

### Impaired islet architecture and endocrine cell composition in IKK2-DN^Pdx1^ mice

**Figure 3:**
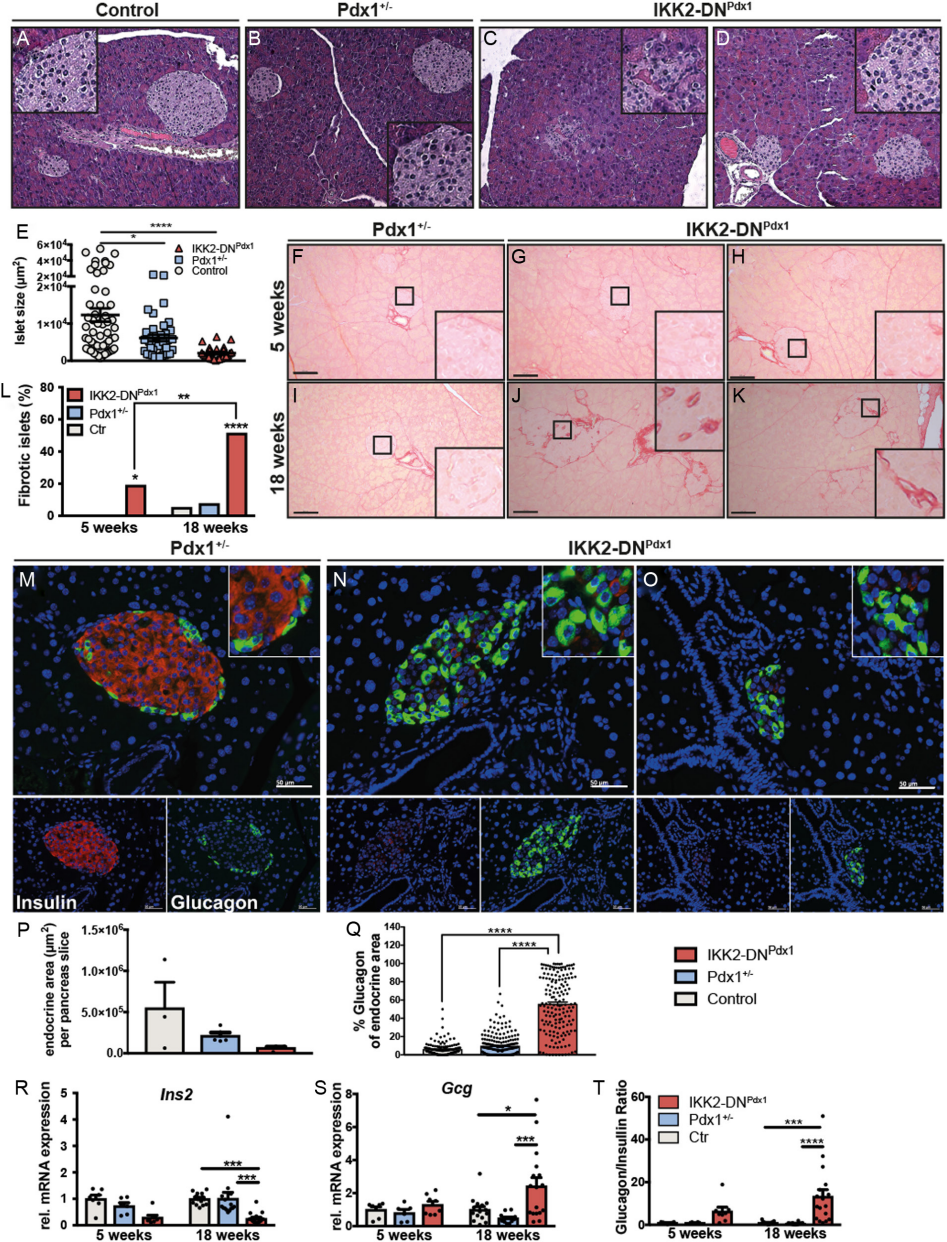
IKK2-DN^Pdx1^ mice develop a prominent, non-immune-mediated type 2 diabetes-like phenotype. Hematoxylin-eosin (HE) staining from pancreas of control (A), Pdx1^+/−^ (B) and IKK2-DN^Pdx1^ (C and D) mice. Quantification of islet size (E) in 12-week-old IKK2-DN^Pdx1^ and control animals (*n* = 2–3 animals; *n* = 28–59 islets). Representative pictures of Sirius red-stained pancreatic sections of Pdx1^+/−^ (F and I) and IKK2-DN^Pdx1^ (G, H, J, K) mice at the age of 5 (F, G and H) and 18 (I, J and K) weeks (scale bar 100 µm). Quantification of fibrotic islets (L). (5 weeks: control *n* = 4, Pdx1^+/−^ *n* = 2 and IKK2-DN^Pdx1^ *n* = 4 animals; *n *= 28–65 islets; 18 weeks: control *n* = 2, Pdx1^+/−^ *n* = 4 and IKK2-DN^Pdx1^ *n* = 5 animals; *n* = 59–81 islets.) Statistical significance of contingency tables was determined by Fisher’s exact test. Immunofluorescence staining of pancreatic sections shows insulin-positive cells (red) and glucagon-positive cells (green) in islets of IKK2-DN^Pdx1^ (N, O) mice compared to Pdx1^+/−^ mice (M) at the age of 18 weeks (scale bar 50 µm). Quantification of endocrine area (P, total insulin- and glucagon-positive area/pancreatic slice in µm^2^) and the proportion of glucagon-positive area (Q,%). Pancreatic slices (*n* = 3 for controls, *n* = 5/group for Pdx1^+/−^ and IKK2-DN^Pdx1^ animals) with a minimum of 20 islets/slice were used for evaluation. Expression of insulin (R) and glucagon (S) in isolated islets was assessed by quantitative RT-PCR in 5 (control *n* = 7, Pdx1^+/−^ *n* = 6, IKK2-DN^Pdx1^ *n* = 8) and 18 (control *n* = 15, Pdx1^+/−^ *n* = 15, IKK2-DN^Pdx1^ *n* = 17) week old animals as indicated. The resulting glucagon/insulin ratio (T). Hprt was used as a reference gene. Results were normalized to control littermates and analysed by one-way (E, P and Q) or two-way (L, R, S and T) ANOVA followed by Bonferroni post-test. (E, L, P, Q, R, S and T) Results are presented as mean ± s.e.m. *P < 0.05; **P < 0.01; ***P < 0.001; ****P < 0.0001.

Having shown that β-cell-specific IKK/NF-κB inhibition in MODY4 mice promotes severe diabetes development we further characterized the diabetic phenotype of IKK2-DN^Pdx1^ mice by histological analyses. H&E staining revealed a prominent destruction in the architecture of individual islets in diseased IKK2-DN^Pdx1^ mice (Fig. 3A, B, C and D) a reduction of the mean islet size (Fig. 3E) and an overall decrease in the chromogranin A-positive area and islets in 16-week-old animals (Supplementary Fig. 3A, B and C). The altered islet morphology of IKK2-DN^Pdx1^ mice was not accompanied with any obvious infiltration of immune cells (Supplementary Fig. 3D). Interestingly, we detected islet fibrosis in IKK2-DN^Pdx1^ mice and the extent increased with disease progression as detected by Sirius red staining (Fig. 3F, G, H, I, J, K and L).

Immunofluorescence staining of pancreatic slices from 16- to18-week animals revealed that islets of control and Pdx1-tTA mice exhibit a typical architecture with a core of insulin-positive β-cells surrounded by a rim of glucagon-positive α-cells (Fig. 3M). In contrast, IKK2-DN^Pdx1^ mice showed a strong reduction in insulin-positive cells, while the number of glucagon-positive cells was increased, and the cells were evenly spread over the whole islet (Fig. 3N and O). Further, although the overall endocrine area (α- and β-cell area) of IKK2-DN^Pdx1^ mice was diminished compared to Pdx1^+/−^ littermates (Fig. 3P), the area expressing glucagon was increased to up to 60% of the endocrine area (Fig. 3Q). The expression of somatostatin was not altered (Supplementary Fig. 3E). As a consequence, plasma insulin levels were reduced in 18-week-old IKK2-DN^Pdx1^ mice compared to 5–8-week-old animals and control littermates (Supplementary Fig. 3F). Quantitative RT-PCR analysis of isolated islets confirmed the reduction in insulin mRNA (Fig. 3R) and the increase in glucagon expression (Fig. 3S), which was more pronounced in IKK2-DN^Pdx1^ mice at later disease stages. So, the glucagon/insulin ratio massively increased with aging (Fig. 3T). Consistent with this IKK2-DN^Pdx1^ mice showed elevated plasma glucagon levels (80.5 vs 197.9 pg/mL; Supplementary Fig. 3G).

### NF-κB/Nemo deficiency triggers diabetes development only in Pdx1^+/−^ mice

**Figure 4:**
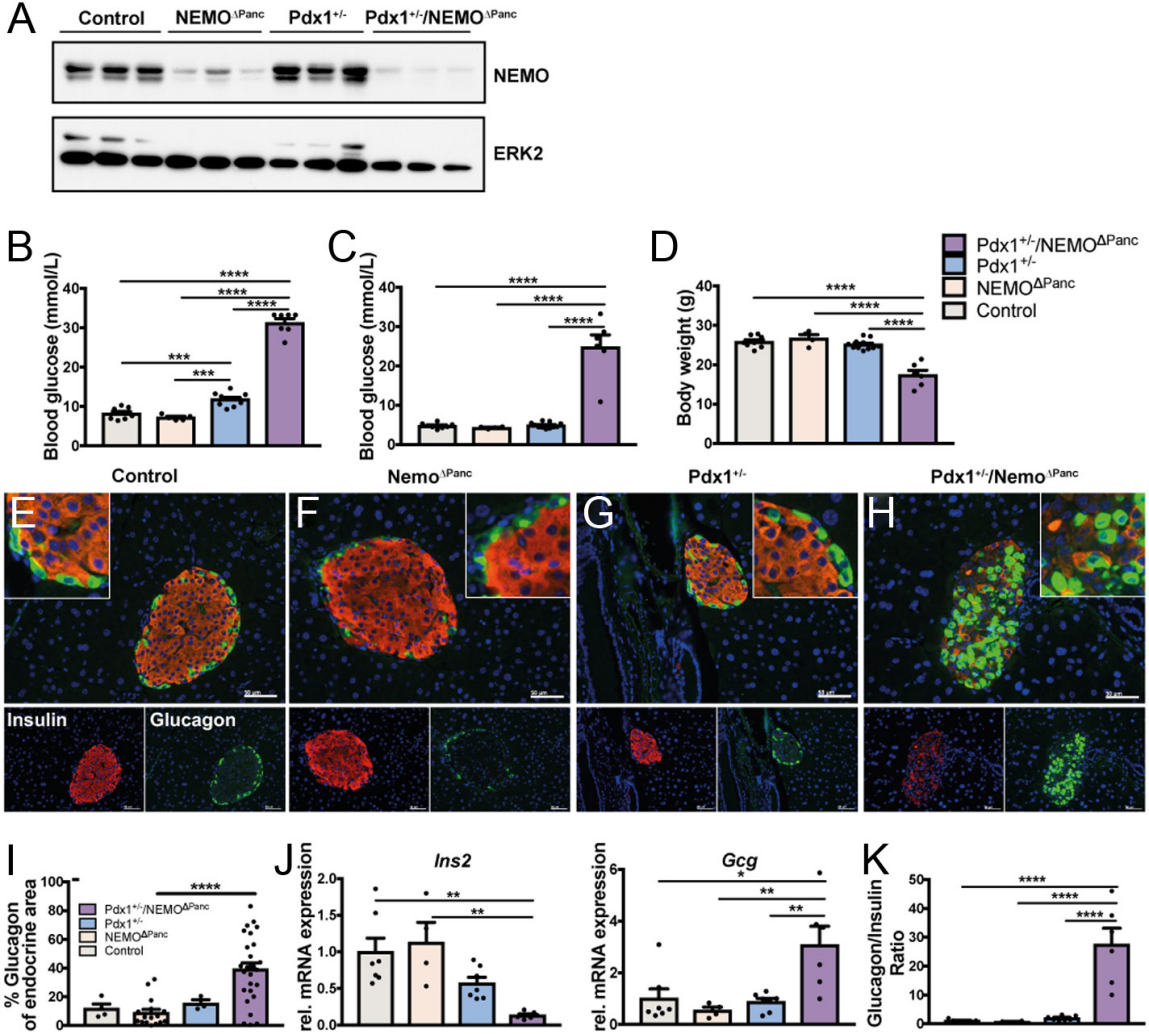
Combination of Pdx1 haploinsufficiency and NEMO deficiency induces diabetes. Western blot analysis indicates successful deletion of NEMO in the pancreas (A) of Pdx1^+/−^/NEMO^ΔPanc^. Fed (B) and fasted (C) blood glucose levels as well as body weight (D) of Pdx1^+/−^/NEMO^ΔPanc^ (*n* = 7) animals (age 16 weeks) compared to control (*n* = 8), NEMO^ΔPanc^ (*n* = 5) and Pdx1^+/−^ (*n* = 11). Immunofluorescence staining for insulin (red) and glucagon (green) of pancreas sections at the age of 16 weeks (E, F, G and H, scale bar 50 µm). Quantification of the glucagon-positive area (I, % of total α + β-cell area). (Control *n* = 3, NEMO^ΔPanc^ *n* = 16, Pdx1^+/−^ *n* = 3 and Pdx1^+/−^/NEMO^ΔPanc^ *n* = 26) Quantitative RT-PCR for insulin and glucagon (J, control *n* = 7, NEMO^ΔPanc^ *n* = 4, Pdx1^+/−^ *n* = 11, Pdx1^+/−^/NEMO^ΔPanc^ *n* = 6). Hprt was used as a reference gene and normalized to control animals. The glucagon/insulin ratio (K). Statistical significance was assessed using one-way ANOVA followed by Bonferroni post-test. Results are presented as mean ± s.e.m. *P < 0.05; **P < 0.01; ***P < 0.001; ****P < 0.0001.

To confirm that NF-κB pathway repression and the subsequent elevation of β-cell stress cooperates with Pdx1 haploinsufficiency in diabetes development, we generated an independent mouse model. We had previously demonstrated that pancreas-specific deletion of Nemo, an essential regulator of NF-κB by itself does not result in diabetes development (Maier *et al.* 2013). This NEMO^ΔPanc^ model was now combined with Pdx-tTA mice to generate the Pdx1^+/−^/Nemo^ΔPanc^ model with MODY4 genotype. Successful deletion of Nemo was confirmed by immunoblot analysis (Fig. 4A). Mice with Pdx1^+/−^ genotype became slightly hyperglycaemic in the fed state (Fig. 4B), which might be attributed to the different genetic background. Notably, Pdx1^+/−^/Nemo^ΔPanc^ mice exhibited strongly increased blood glucose levels in both fed (Fig. 4B, 31.3 ± 1.0 mmol/L) and fasted (Fig. 4C, 27.6 ± 1.6 mmol/L) states, whereas control littermates and mice with Nemo deletion in β-cells but without Pdx1 haploinsufficiency remain inconspicuously. In addition, Pdx1^+/−^/Nemo^ΔPanc^ mice developed typical clinical signs of diabetes like polydipsia, polyuria and reduction of body weight (Fig. 4D). A decrease in insulin-positive cells and an increase in glucagon-positive cells was detected upon immunohistological analysis of Pdx1^+/−^/Nemo^ΔPanc^ mice (Fig. 4F, G, H and I). The glucagon-positive cells represented around 40% of the overall endocrine area (Fig. 4I). The total endocrine compartment was not markedly changed in Pdx1^+/−^/Nemo^ΔPanc^ mice as Chromgranin A expression did not differ strongly between groups (Supplementary Fig. 3H). Accordingly, mRNA expression of insulin was reduced, whereas glucagon mRNA was upregulated in Pdx1^+/−^/Nemo^ΔPanc^ mice (Fig. 4J), thereby resulting in a prominent shift of the glucagon/insulin ratio similar to the IKK2-DN^Pdx1^ model (Fig. 4K). As a consequence plasma glucagon levels of Pdx1^+/−^/NEMO^ΔPanc^ mice were higher compared to Pdx1^+/−^ animals (89.1 vs 185.1 pg/mL; Supplementary Fig. 3I).

### Gene expression profiling of IKK2-DN^Pdx1^ islets reveals limited β-cell functionality, ongoing fibrosis and critical changes in stress responses

To gain insight into the molecular mechanisms that accelerate diabetes development upon NF-κB inhibition in our MODY4 model, we performed microarray-based gene expression analyses using isolated islets of early and progressed disease phases. At the age of 5 weeks, over 400 genes were found deregulated in IKK2-DN^Pdx1^ animals and this number rose to 2600 genes in 18-week-old mice (≥1.5-fold change). Pathway analysis (REACTOME) revealed prominent changes in genes involved in the regulation of β-cell development and function together with insulin secretion (Supplementary Table 3). Insulin expression and marker genes indicative for mature β-cells like *Slc2a2*, *Ucn3* and *Slc30a8* were markedly downregulated in IKK2-DN^Pdx1^ islets. Remarkably, a clear decline was detected in some of these β-cell maturity genes (e.g. *Insrr* and *G6pc2*) from 5 to 18 weeks of age, reflecting disease progression and increase in severity with age. Interestingly, at least 12 genes including *SLC2A2*, *G6PC2*, *SLC30A8*, *PCSK1* and *RAPGEF4*, also overlap with previously implicated genes from genome wide association studies (GWAS) for type 2 diabetes mellitus and blood glucose loci (Supplementary Table 3).

Additionally, transcription factors like *Neurod1* and *Mafb* as well as other genes expressed during pancreatic development were found affected suggesting an alteration of β-cell differentiation factors during disease progression. Furthermore, cell-cycle checkpoint genes as well as genes implicated in cellular senescence were downregulated, further supporting impaired β-cell homeostasis in IKK2-DN^Pdx1^ mice. At the late disease state, an elevated expression of genes involved in ROS detoxification (e.g. *Hmox1*, *Cybb*) became apparent possibly indicating the activation of genetic programs counteracting ER stress due to NF-κB inhibition. Notably, especially in 18-week-old animals genes involved in UPR were mainly downregulated (e.g. *Wfs1*, *Atf3*). To confirm increased β-cell stress levels in in IKK2-DN^Pdx1^ we determined the status of typical stress markers in isolated islets by immunoblot analyses. We found an upregulation of the heat shock protein Hsp27, hemoxygenase 1 (Hmox1) and Atf6 only in IKK2-DN^Pdx1^ samples indicating that the combination of Pdx1 haploinsufficiency with NF-κB inhibition triggers cellular stress including ER and oxidative stress (Supplementary Fig. 4A, B, C and D). As a consequence of enhanced oxidative stress we detected increased nuclear 8-oxoguanine staining in IKK2-DN^Pdx1^ islets (Supplementary Fig. 4E, F, G, H, I, J, K, L and M).

Consistent with the increased islet fibrosis, the expression of numerous genes involved in extracellular matrix (ECM) organization (e.g. various matrixmetalloproteases, integrins, fibronectin, laminin, cathepsins) was significantly elevated at advanced disease state (Supplementary Table 3). Interestingly, the highest upregulated genes included Cholecystokinin (*Cck*) and serine proteases like *Gm10334* and *Prss3* that belong to the trypsin family (Alloy *et al.* 2015). So far these serine proteases have not been immediately linked to diabetic conditions.

**Figure 5:**
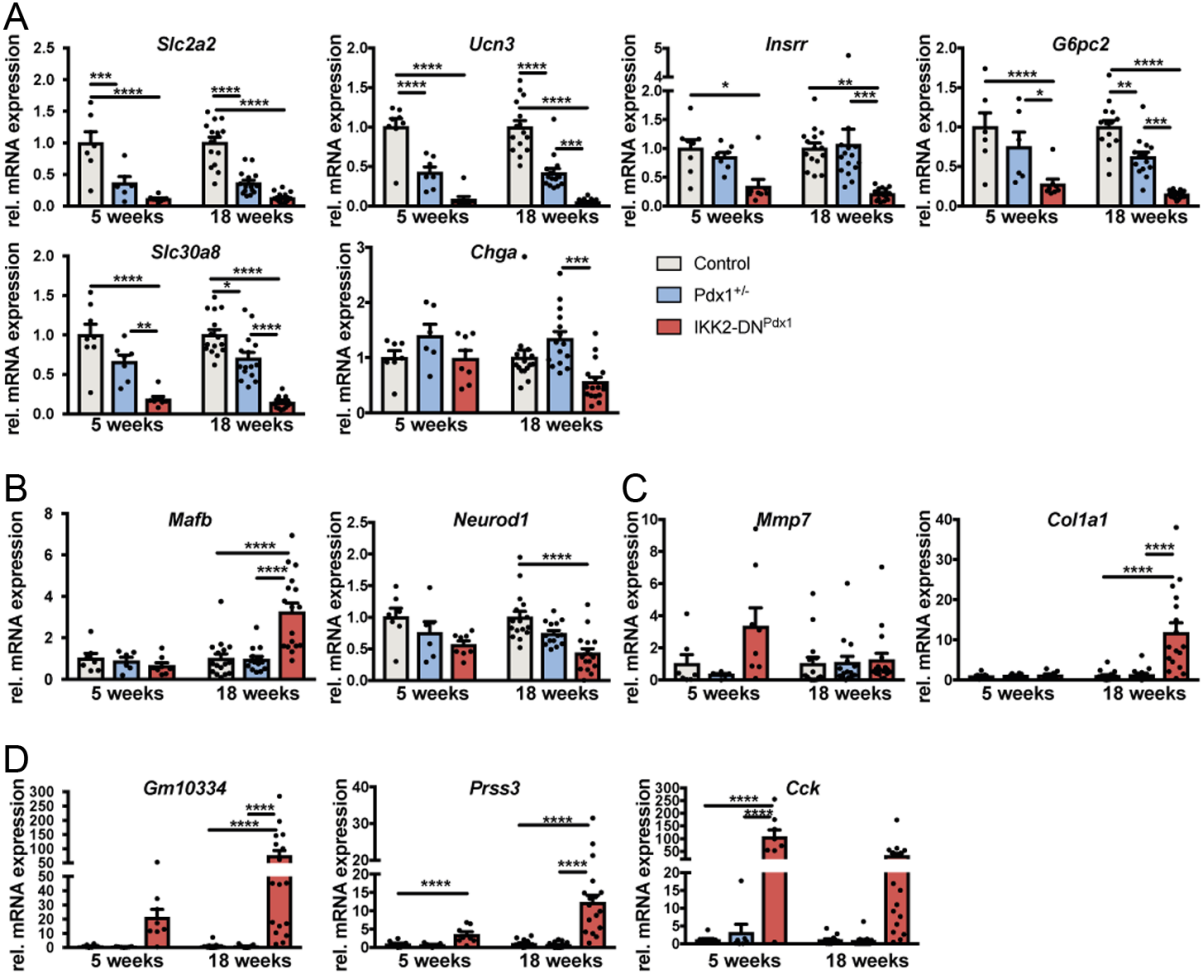
Expression of candidate genes is strongly deregulated in IKK2-DN^Pdx1^ mice upon diabetes development. Expression of selected genes identified in the microarray-based gene expression profiling was validated by qRT-PCR from isolated islets of 5- and about 18-week-old animals as indicated. Deregulated expression of genes preserving β-cell function (A), transcription factors regulating β-cell development (B), ECM-modulating genes (C) as well as serine proteases and *Cck* (D) is depicted as indicated. Relative expression of the genes was compared to control littermates (at 5 weeks of age, control *n* = 7, Pdx1^+/−^ *n* = 6, IKK2-DN^Pdx1^ *n* = 8; at 18 weeks of age, control *n* = 15, Pdx1^+/−^ *n* = 15, IKK2-DN^Pdx1^ *n* = 17). Hprt was used as a reference gene. Results were analysed by two-way ANOVA analysis followed by Bonferroni post-test and presented as the mean ± s.e.m. *P < 0.05; **P < 0.01; ***P < 0.001; ****P < 0.0001.

Selected genes were further validated via qRT-PCR including additional samples (Fig. 5). These gene expression data further confirmed the marked changes in β-cell function, homeostasis and identity in the IKK2-DN^Pdx1^ model (Fig. 5A and B). Notably, a significant reduction in expression of genes associated with β-cell function (e.g. *Slc2a2*, *Ucn3*, *G6pc2*, *Slc30a8*; Fig. 5A) was already detectable in islets of Pdx1^+/−^ mice indicating the pre-dysfunctional state of Pdx1^+/−^ β-cells. Indicative for the prominent islet fibrosis in IKK2-DN^Pdx1^ mice we found strong expression of the matrix metalloprotease gene *Mmp7* at early disease state, whereas type1 collagen gene, *Col1a1*, was upregulated upon disease progression (Fig. 5C). The serine proteases and *Cck* showed prominent expression at both early and late phases of diabetes development (Fig. 5D).

### Severe MODY4 diabetes can be reversed in IKK2-DN^Pdx1^ mice

**Figure 6:**
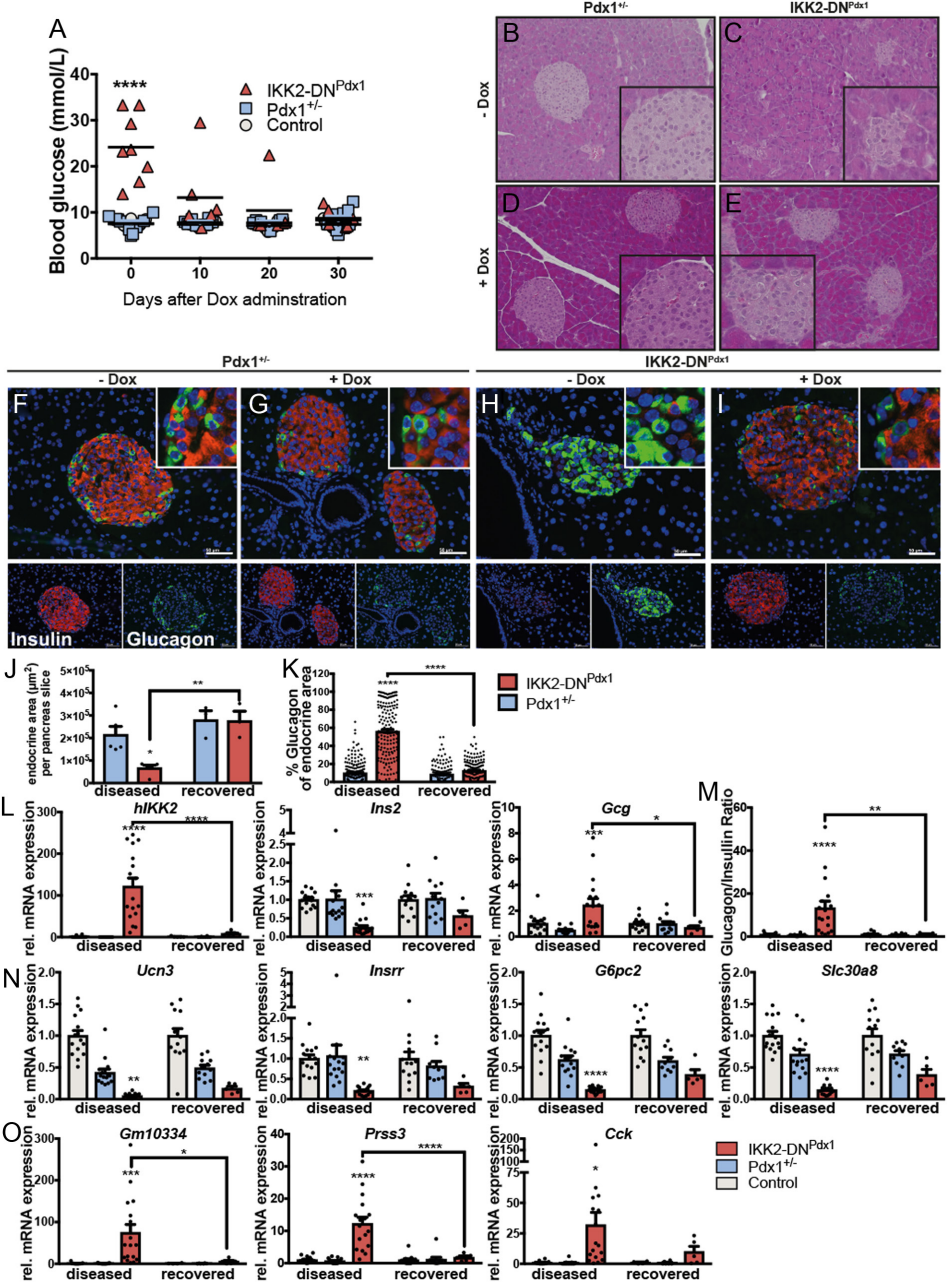
ER stress-induced diabetes is reversible in MODY4 animals. To inactivate transgene expression mice at the age of 16 weeks received Dox (1 g/L) in the drinking water for 30 days. Fed blood glucose levels before (0) and during Dox application (A) are depicted (one-way ANOVA including Bonferroni correction). Hematoxylin-eosin (B, C, D and E) and immunofluorescence staining (F, G, H and I) of paraffin sections from IKK2-DN^Pdx1^ mice before (B, C, F and H) and after Dox administration (D, E, G and I) insulin-positive cells (red) and glucagon-positive cells (green, scale bar 50 µm). Quantification of endocrine area (J, total insulin- and glucagon-positive area/pancreatic slice in µm^2^) and the proportion of glucagon-positive area (K, %) before (diseased) (*n* = 5/group) and after Dox administration (recovered) (*n* = 3/group) animals. qRT-PCR from isolated islets (L, M, N and O). Relative expression of the *hIKK2* transgene, insulin (*Ins*) and glucagon (*Gcg*) in IKK2-DN^Pdx1^ mice compared to control and Pdx1^+/−^ mice (L) and the resulting glucagon to insulin ratio (M). Expression of genes necessary for physiological β-cell function (N, Ucn3, Insrr, G6pc2, Slc30a8). The expression of serine proteases and *Cck* (O). Shown is relative mRNA expression as indicated (in diseased state, control *n* = 15, Pdx1^+/−^ *n* = 15, IKK2-DN^Pdx1^ *n* = 17; in recovered state, control *n* = 13, Pdx1^+/−^ *n* = 12, IKK2-DN^Pdx1^ *n* = 5). Hprt was used as a reference gene and results normalized to control animals. Results were analysed by two-way ANOVA analysis followed by Bonferroni post-test. (A, D, E, F, G and H) Results are presented as the mean ± s.e.m. *P < 0.05; **P < 0.01; ***P < 0.001; ****P < 0.0001.

To examine whether the severe diabetic phenotype is dependent on continuous stress insults, we restored normal IKK/NF-κB signalling in diabetic IKK2-DN^Pdx1^ mice by Dox administration to switch off transgene expression and to normalize the cell stress response. The initial diabetic condition was confirmed by fed blood glucose values that ranged from 13.9 mmol/L to 33.3 mol/L prior to Dox treatment (Fig. 6A). Already 10 days after Dox application blood glucose levels decreased and most mice showed normalization of blood glucose after 30 days of Dox treatment (Fig. 6A). Dox-dependent transgene inactivation was confirmed by Western blot (Fig. 1A) and qRT-PCR (Fig. 6L). Interestingly, Dox-treated IKK2-DN^Pdx1^ mice (Fig. 6E) regained islet architecture similar to that of Pdx1^+/−^ animals (Fig. 6B and D). Immunostaining also demonstrated the reappearance of β-cell-rich islets, which strongly stained for insulin (Fig. 6F, G, H and I). Furthermore, the endocrine area increased (Fig. 6J) and the high number of glucagon-positive cells found in diabetic IKK2-DN^Pdx1^ mice decreased again to normal levels (Fig. 6K). In line with islet recovery, insulin expression considerably increased after transgene inactivation, whereas glucagon expression returned to control levels (Fig. 6L); thus, insulin/glucagon ratio became normalized (Fig. 6M). Moreover, gene expression markers for mature β-cells (*Ucn3*, *Insrr*, *G6pc2*, *Slc30a8*) increased again (Fig. 6N) while serine proteases (*Gm10334*, *Prss3*) upregulated in diseased animals returned to control levels and *Cck* expression also decreased again (Fig. 6O). Taken together our data clearly indicate that the β-stress-dependent acceleration of severe type 2 diabetes development in otherwise glucose-intolerant MODY4 mice is a reversible process.

### β-Cell proliferation participates in the reversion of diabetes in MODY4 mice

**Figure 7:**
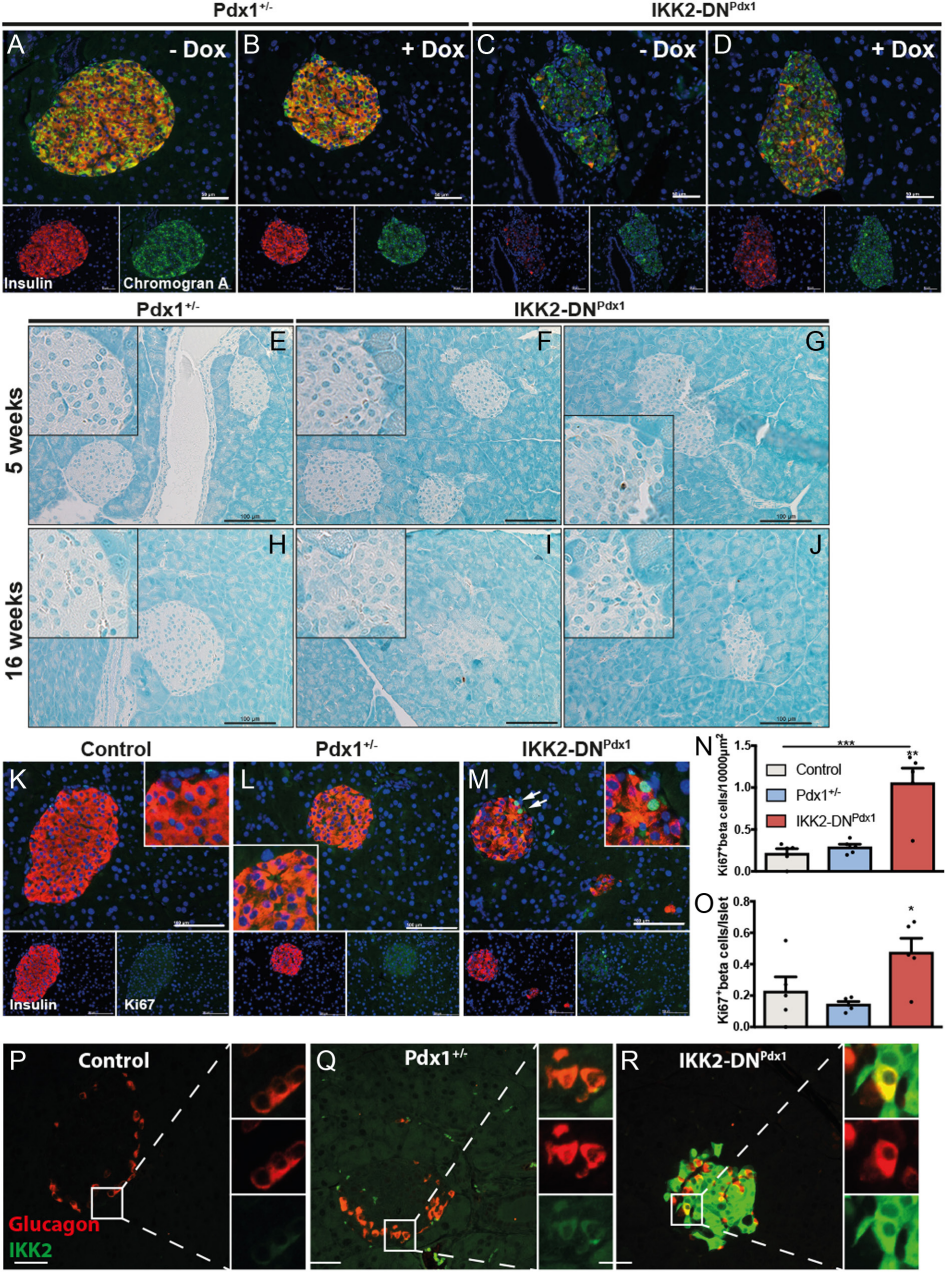
Cellular differentiation and proliferation is involved in diabetes recovery in MODY4 mice. Mice received Dox to inactivate transgene expression until blood glucose decreased to moderate levels of ~16.7 mmol/L (A, B, C, D and K, L, M, N). Immunofluorescence staining (A, B, C and D) of insulin (red), the pan-endocrine marker chromogranin A (green) and DAPI (blue, scale bar 50 µm). Apoptosis analysis by TUNEL assay of pancreatic sections from 5 (E, F and G) and 16 (H, I and J) weeks old Pdx1^+/−^, and IKK2-DN^Pdx1^ mice. Sections were counterstained with methyl green (scale bar 100 µm). Insulin (red), Ki67 (green), and DAPI (blue) co-staining of pancreatic sections from Pdx1^+/−^ and IKK2-DN^Pdx1^ mice (L, M, scale bar 100 µm) Ki67-positive β-cells (white arrows) were quantified (N, O; *n* = 5/group, 137-200 islets/group). (P, Q and R) Representative pictures of pancreatic islets stained for glucagon (red) and IKK2 (green) from 5-week-old control, Pdx1^+/−^ and IKK2-DN^Pdx1^ animals (scale bar 50 µm).

To get more information on the mechanisms accounting for the fast recovery of insulin-positive cells in diabetic IKK2-DN^Pdx1^ animals, we analysed the expression of the pan-endocrine marker chromogranin A together with insulin during the recovery phase (Fig. 7A, B, C and D). Interestingly, although slightly reduced in diseased IKK2-DN^Pdx1^ mice, the whole islet stained positive for chromogranin A, while insulin was virtually undetectable (Fig. 7C), but reappeared under Dox treatment (Fig. 7D). This together with the reduced expression of β-cell maturity genes suggest that stress-induced diabetes development in the IKK2-DN^Pdx1^ model is accompanied by the loss of β-cell identity as evidenced by loss of insulin expression. Upon discontinuation of the stress insult and revival of IKK/NF-κB signalling, cells with β-cell identity reappeared as measured by the restoration of chromogranin A and insulin double-positive cells. TUNEL staining at the age of 5 (Fig. 7E, F and G) and 16 (Fig. 7H, I and J) weeks revealed that disease development and alteration in islet architecture was not attributed to massively elevated cell death.

Interestingly, the number of Ki67-immunoreactive β-cells increased during reversion of the diabetic phenotype (Fig. 7K, L, M, N and O), suggesting that the recovery process includes β-cell proliferation. The observed proliferation may contribute to the reappearance of the endocrine area in normoglycaemic IKK2-DN^Pdx1^ animals after 30 days of recovery (Fig. 6J and K). Whether IKK2-DN expression in haploinsufficient and insulin-positive β-cells initiates dedifferentiation processes to force glucagon and suppress insulin expression in these cells remains unclear. We identified only isolated, double-positive cells with glucagon and IKK2-DN transgene expression, which would support dedifferentiation events in our model (Fig. 7P, Q and R).

## Discussion

In this study we demonstrated that enhanced β-cell stress induced by IKK/NF-κB inhibition aggravates an otherwise indolent moderate hyperglycaemia and induces progressive form of diabetes in a mouse model of MODY4. Remarkably, this pronounced diabetic phenotype returned to normoglycemia when the β-cell stressor was set aside. These findings identify augmented β-cell stress as a critical condition that is able to worsen the course of MODY4 to an early-onset type 2 diabetes-like phenotype. Our model system offers now a possible explanation for the heterogenic clinical situation found in families where mutation carriers are predisposed to phenotypes of mild MODY or severe type 2 diabetes (Gragnoli *et al.* 2005, Fajans *et al.* 2010, Fajans & Bell 2011, Doddabelavangala Mruthyunjaya *et al.* 2017). So far it is unclear to what extent environmental challenges contribute to the specific individual metabolic phenotype development of patients carrying similar mutations. However, pregnancy-related metabolic β-cell stress was reported to increase the prevalence of diabetes development in MODY patients (Gjesing *et al.* 2017). Another factor associated with β-cell stress is obesity, which is able to trigger diabetes development at young age in MODY4 mutation carriers (Weintrob *et al.* 2008). In other MODY subtypes the influence of BMI on disease development remains open. The specific mechanisms by which obesity aggravates disease progression in MODY4 are not defined but elevated ER stress due to β-cell compensation in insulin-resistant states seems plausible (Cnop *et al.* 2017). In support of this hypothesis, Pdx1 haploinsufficiency limits the compensatory islet hyperplastic response in different mouse models of insulin resistance and results in prominent diabetes development indicating a specific stress sensitivity of Pdx1 hemizygous β-cells (Kulkarni *et al.* 2004). It remains to be clarified to what extent elevated β-cell stress levels promote disease development in cases of polygenic type 2 diabetes. However, epigenetic modifications of *PDX1* reducing its activity could contribute to the development of type 2 diabetes since this transcription factor binds to the promoter of a large set of genes related to β-cell survival as well as insulin secretion. (Khoo *et al.* 2012, Yokoi 2017). Accordingly, we detected a further decrease in *Pdx1* expression with disease progression.

Consistent with previous data (Ahlgren *et al.* 1998) Pdx1.tTA knock-in mice used in our study remain healthy aside from glucose intolerance thereby phenocopying the mild disease form observed in some MODY4 patients. However, as previously stated (Johnson *et al.* 2003), some 35-week-old Pdx1.tTA mice exhibited high blood glucose levels up to 26.6 mmol/L (data not shown) indicating that aging is able to trigger diabetes development in Pdx1^+/−^ animals. However, in our conditional IKK2-DN^Pdx1^ model this process is strongly accelerated and triggers an early-onset type 2 diabetes phenotype.

Previously, we have shown that constitutive activation of IKK/NF-κB signalling in β-cells leads to the initiation of a typical form of immune-mediated diabetes (Salem *et al.* 2014). IKK2-DN^Pdx1^ mice develop a non-immune diabetic phenotype, which however is associated with prominent islet fibrosis and gene expression changes indicative for massive ECM reorganization and thereby phenocopying to some extent the pathology found in type 2 diabetes patients (Lee *et al.* 2017). IKK2-DN^Pdx1^ mice are non-obese and non-hyperinsulinaemic, and similar to AKITA mice they exhibit a reduction of their β-cell mass without insulitis and develop islet fibrosis like Torri rats (Srinivasan & Ramarao 2007, King 2012).

Oxidative stress and ER stress are common forms of β-cell stress occurring in type 2 diabetes initiated by obesity or aging (Henriksen *et al.* 2011, Asmat *et al.* 2016). NF-κB signalling is able to induce expression of genes with antioxidative activities to escape ROS-induced cell stress (Morgan & Liu 2011). Indeed, pathway analysis of deregulated genes in diseased IKK2-DN^Pdx1^ mice showed a clustering of genes that are involved in ROS and ER stress regulation. Although there was upregulation of ROS-producing genes like *Cybb*, other genes (e.g. *Hmox1*) responsible for ROS clearance were also expressed. This might indicate that IKK/NF-κB inhibition overall deregulates the balance between stress-promoting and stress-protecting mechanisms in Pdx1 haploinsufficient β-cells. Pdx1^+/−^ β-cells have an increased susceptibility to ER stress and high-fat diet imposes increased ER stress to β-cells of Pdx1^+/−^ mice leading to overt diabetes (Sachdeva *et al.* 2009). In our model, ER stress is aggravated upon impairment of NF-κB signalling that is reflected by marked downregulation of genes associated with UPR at progressed disease states. This includes *Wfs1* (Wolfram syndrome 1), a typical factor involved in UPR located in the ER. Patients with mutated *WFS1* suffer from childhood-onset diabetes and optic atrophy (Bansal *et al.* 2018, Barrett & Bundey 1997). Interestingly, both patient-derived WFS1-deficient β-cells (Shang *et al.* 2014) and mice lacking *Wfs1* in β-cells (βWfs^−/−^) show increased ER stress, which is associated with β-cell dysfunction and diabetic phenotype development (Riggs *et al.* 2005). Similar to the phenotype of IKK2-DN^Pdx1^ animals, βWfs^−/−^ mice exhibited significantly disrupted islet architecture and an altered ratio of β-cells to non- β-cells within the islets (Riggs *et al.* 2005), whereas Pdx1^+/−^ mice show only mild changes with aging. Notably, postmortem analysis of patients with Wolfram syndrome revealed a non-immune type destruction of islets similar to the IKK2-DN^Pdx1^ phenotype (Karasik *et al.* 1989).

Using two independent mouse models, we could demonstrate that inhibition of IKK/NF-κB signalling results only in a severe, early-onset diabetic phenotype in the context of a MODY4 genotype. The first model uses the expression of a dominant-negative allele of IKK2, while the second one bases on the deletion of Nemo in β-cells. Previous studies in mice with repressed/blocked IKK/NF-κB signalling in β-cells with two functional *Pdx1* alleles revealed either no change in blood glucose homeostasis, islet morphology and function (Eldor *et al.* 2006, Maier *et al.* 2013) or in one study a very mild increase in fed blood glucose levels (Norlin *et al.* 2005). In the latter case NF-κB was proposed to maintain glucose-stimulated insulin secretion. Gene expression profiling data of our model emphasized the importance of NF-κB in the regulation of the ER stress response and β-cell maintenance under MODY4 conditions. This was manifested by upregulation of UPR stress-response and downregulation of β-cell maturation genes upon NF-κB inhibition. Indeed, our data support the idea that Pdx1 and IKK/NF-κB signalling functionally cooperate to maintain normal β-cell homeostasis and to cope with stress normally occurring in β-cells.

The late occurring reduction in chromogranin A-positive area suggests that prolonged stress initiates the overall loss of endocrine cells although we could not detect a high level of TUNEL-positive cells, neither in 5-week-old nor in 16-week-old animals. The size of individual islets is reduced in IKK2-DN^Pdx1^ mice, which might be a consequence of reduced compensatory β-cell proliferation under chronic stress conditions. Accordingly, we observed decreased mRNA levels of cell-cycle checkpoint genes together with an increased expression of genes involved in the modulation of cellular senescence. Collectively, these findings suggest that the loss of β-cell identity manifested by a dramatic reduction in insulin expression together with a dysfunctional secretory response precedes overall endocrine cell loss and is responsible for the switch to full diabetes development in our model system. This principle was discussed as an important mechanism in the development of type 2 diabetes in mice and men and was associated by an increase of the α/β-cell ratio (Cinti *et al.* 2016, Bensellam *et al.* 2017). In line with that, we detected an increase in glucagon-positive cells and a decrease in insulin-positive cells. So far, it is an open question whether MODY4 mutation carriers suffering from early-onset type 2 diabetes also show this change in α/β-cell ratio.

A great advantage of our novel MODY4 animal model is its reversibility, which allows to model mild and severe disease courses of MODY4 and to address mechanisms of diabetic phenotype reversion. Interestingly, we could show that transgene inactivation normalizes expression of genes necessary for β-cell maintenance and function. Even more important, blood glucose levels were restored, evidencing that after elimination of the stressor, MODY4 β-cells are able to regain normal functionality. The increased number of Ki67-positive β-cells during Dox administration implies that proliferation contributes, at least partially, to diabetes recovery in the IKK2-DN^Pdx1^ model. Also, the normalization of β-cell maturity marker gene expression can be explained by proliferation-based β-cell mass restoration. However, we cannot exclude that re-differentiation events are involved in the functional recovery of β-cells and the reversion of diabetes in IKK2-DN^Pdx1^ mice.

In summary, our conditional model system allows the reversible switch between a mild and a fast progressing form of MODY4, which is highly relevant for the understanding of mechanisms underlying clinical heterogeneity in this disease. Importantly, the current work suggests that avoidance or retraction of chronic β-cell stress could serve as therapeutic strategy to prevent or even reverse detrimental clinical progression in MODY4.

## Supplementary Material

Supplementary Figure 1: Loss-of-function mouse model for conditional inhibition of IKK2 in pancreatic β cells. (A) Transgenic approach for Dox regulated expression of IKK2 DN in pancreatic β cells. The tTA protein is expressed under the control of the endogenous Pdx1 promoter and can bind to the bidirectional (tetO)7 promoter driving transcription of the IKK2 DN transgene as well as the luciferase reporter gene. Dox inhibits binding of tTA to the promoter thereby shutting off transgene expression. The Pdx1.tTA mice are knock in animals in which the coding sequence of tTA has replaced the endogenous Pdx1 gene resulting in haploinsufficiency for Pdx1 in this mouse line (Pdx1+/-). (B) Luciferase reporter gene activity was elevated in the pancreas (Pa) and parts of intestine (Int1-3) of 12 week old IKK2 DNPdx1 mice compared to controls (n=4). In the spleen (SP), liver (Li), lung (Lu), thymus (Thy) and stomach (St) luciferase activity was comparable to controls. (C) Western blot analysis of IKK2 expression in different organs of 12 week old control (-) and IKK2-DNPdx1 mice (+). Extracellular signal-related kinase-2 (ERK2) was used as loading control. IKK2 expression was increased in pancreata and intestine of IKK2 DNPdx1 mice. IKK2 was also detectable in thymus extracts but at comparable levels in IKK2 DNPdx1 and control mice (D) Electrophoretic mobility shift assay of LPS treated islets with extracts using an NF-κB specific probe showing comparable NF κB activity in Pdx1+/- and control mice and reduced NF κB activity after stimulation in IKK2 DNPdx1 mice compared to Pdx1+/- littermates. (E-G) Immunofluorescence staining showing IKK2 DN transgene expression (green) specifically in pancreatic islets. Combined staining with DAPI (blue), (E) insulin (red), (F) glucagon (red) and (G) somatostatin (red) shows co-localization of IKK2 exclusively with insulin-positive cells (E).

Supplementary Figure 2: Analysis of disease progression in IKK2 DNPdx1 mice. (A) Fed and (B) fasted blood glucose levels and (C) body weight of 5 (n=4 8/group), 8 (n=5 9/group), 12 (n=6 9/group) and 16 (n=12 17/group) week old IKK2 DNPdx1 mice and control littermates are shown. Fed blood glucose levels were constantly high in IKK2 DNPdx1 animals, while fasted blood glucose values increased with disease progression. Results were analyzed by two-way (A-C) ANOVA analysis followed by Bonferroni post-test and presented as the mean ± SEM. *: p < 0.05; **: p < 0.01; ***: p < 0.001; ****: p < 0.0001.

Supplementary Figure 3: Islet morphology in IKK2 DNPdx1 mice. (A) Representative pictures of chromogranin A stained pancreata of 5 and 16 week old animals. (B, C) Quantification of total chromogranin A-positive area (% of total pancreatic area/slice, left panel) and islet number (right panel) was assessed by Image J software as indicated. The chromogranin A-positive area and islet number of 5 week old IKK2 DNPdx1 animals is similar to controls (B, n=3-4/group). In 16 week old IKK2 DNPdx1 animals a tendency of reduced chromogranin A-positive area and islet number is detectable. (C, n=4/group). (D) Immunofluorescence staining for insulin (red), CD45 (green) and DAPI (blue) did not show prominent infiltration of CD45-positive immune cells in islets of IKK2 DNPdx1 mice at the age of 18 weeks. (E) Immunofluorescence staining of insulin (red), somatostatin (green) and DAPI (blue) in pancreatic sections of 12 week old IKK2 DNPdx1 mice did not reveal obvious differences between genotypes. (F) Plasma insulin levels at the age of 5-8 (n=4/group) and 18 weeks (n=4 for control and Pdx1+/- animals, n=3 for IKK2-DNPdx1 mice). (G) Plasma glucagon levels at the age of 8-12 weeks (n=7 for control and Pdx1+/- animals, n=9 for IKK2-DNPdx1 mice). (H) Quantitative RT-PCR for chromogranin A (Chga) expression in isolated islets from 12 week old animals (control n=4, NEMOΔPanc n=3, Pdx1+/- n=4, Pdx1+/-/NEMOΔPanc n=6). Hprt was used as a reference gene and normalized to control animals. (I) Plasma glucagon levels at the age of 12 weeks (n=4 for control, n=5 for Pdx1+/- and Pdx1+/-/NEMOΔPanc mice). Results were analyzed by one-way ANOVA analysis followed by Bonferroni post-test and presented as the mean ± SEM. *: p < 0.05; **: p < 0.01; ***: p < 0.001; ****: p < 0.0001.

Supplementary Figure 4: Cellular Stress is increased in islets of IKK2 DNPdx1 mice. (A) Immunoblot of pancreatic islet extracts from 18 week old mice. (B-D) Quantification of protein expression levels for Atf6α, Hmox1 and Hsp27 (n=6/group for control and Pdx1+/-, n=7 for IKK2-DNPdx1 animals). The AUC was calculated using the Image J software and normalized to β-Actin. (E-L) Representative pictures of 8-Oxoguanine (8-OG) stained pancreatic sections of control (E,F) Pdx1+/- (G,H) and IKK2-DNPdx1 (I-L) mice at the age of 5 weeks. (M) Quantification of 8-OG expression levels in pancreatic islets (n=4/group for control and IKK2-DNPdx1, n=2 for Pdx1+/- animals). A minimum of 3 islets/slice were used for evaluation. Statistical significance was assed using one-way ANOVA. Results are presented as mean ± SEM. *: p < 0.05; **: p < 0.01; ***: p < 0.001; ****: p < 0.0001

Supplementary Table 1: Composition of mouse standard chow diet.

Supplementary Table 2: Antibodies used for western immunoblotting, immunohistochemistry and immunofluorescence staining.

Supplementary Table 3: Pathway analysis of deregulated genes identified by microarray-based gene expression profiling of IKK2 DNPdx1 mice at early and later disease phases. Microarray analysis was performed with whole islet mRNA of 5 week and 18 week old mice (n=4 6). Data were analyzed with the Genesifter software using ANOVA analysis followed by Benjamini and Hochberg post-test. Pathway analysis was performed with the REACTOME software taking deregulated genes into account with a log2 fold change ≥ 1.5 compared to Pdx1+/- animals at 5 or 18 weeks of age. Selected pathways associated with β-cell dysfunction and cellular stress are depicted. qRT-PCR validated genes are underlined. Genes associated with T2D loci as identified by GWAS Catalog and FUMA are indicated.

## Declaration of interest

The authors declare that there is no conflict of interest that could be perceived as prejudicing the impartiality of the research reported.

## Funding

This work was supported by grants from the Boehringer Ingelheim Ulm University BioCenter (BIU-C6) and the GRK-1041(P3) to B B. B O B was supported by Lee Kong Chian School of Medicine, NTU, Singapore start-up-grant.

## Author contribution statement

B T, H H S, T W and B B designed and wrote the manuscript. B T, H H S, L L and B B performed research and analyzed data. R D performed bioinformatic analyses. L L, H N, E S, M W and B O B designed the study, revised and edited the manuscript. B B is the guarantor of this work and, as such, had full access to all the data in the study and takes responsibility for the integrity of the data and the accuracy of the data analysis.
