# Supplementary figures and images for: Elevated β-cell stress levels promote severe diabetes development in mice with MODY4
Source: J Endocrinol. 2019 Nov 4;244(2):323–37. doi: 10.1530/JOE-19-0208 (PMC6933809; doi:10.1530/JOE-19-0208)

**A**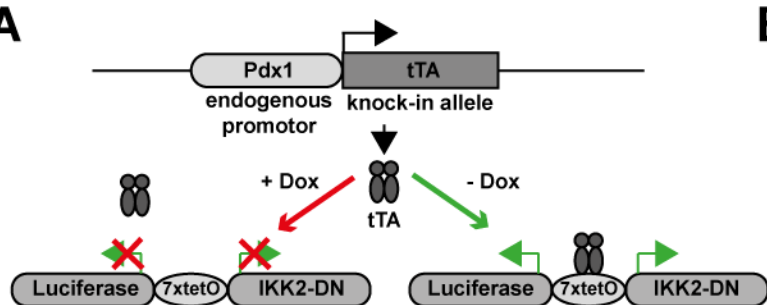**B**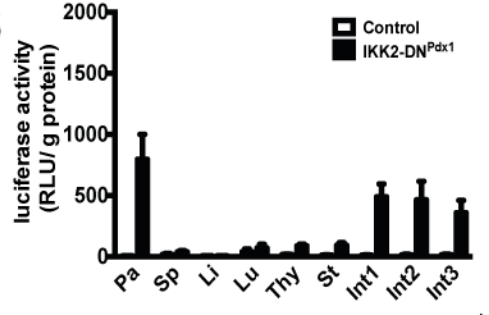**C**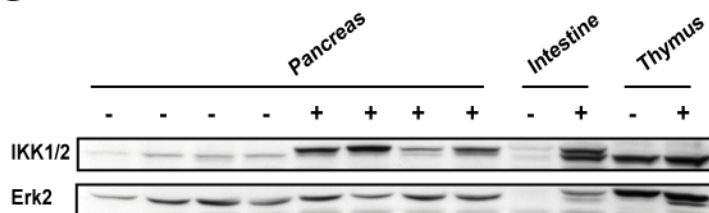**D**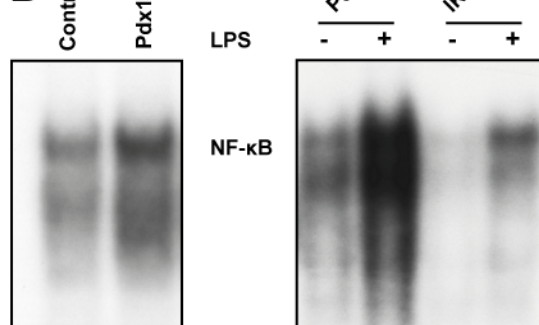**E**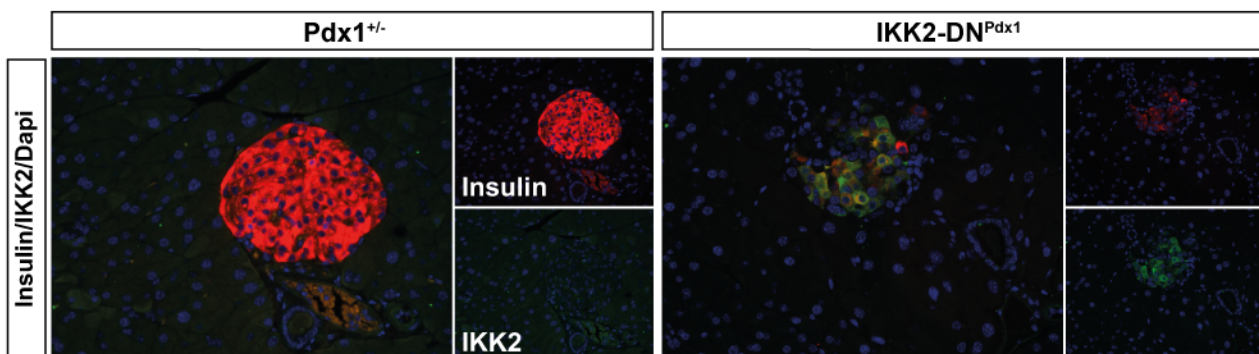**F**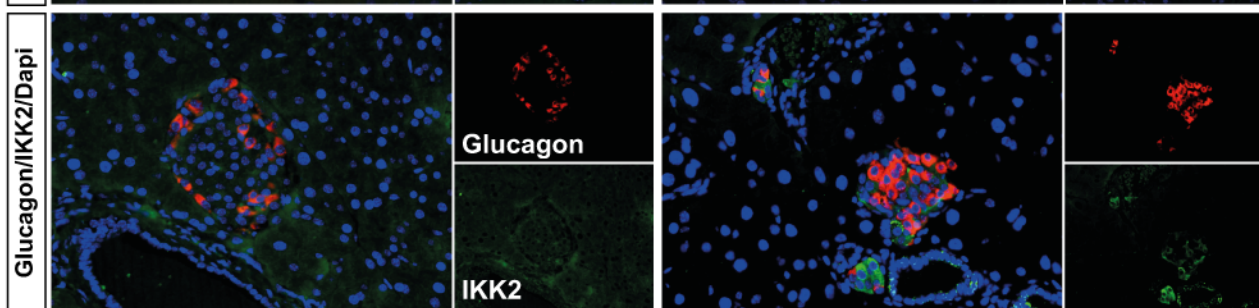**G**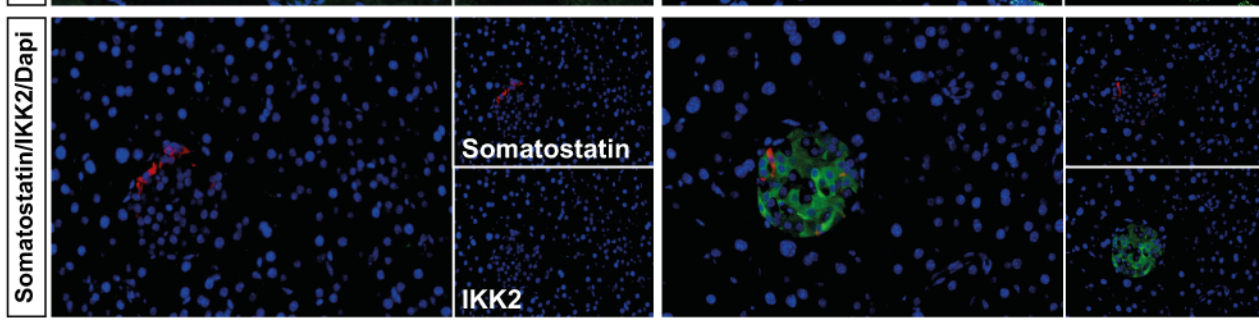

Supplement: Supplementary Figure 1: Loss-of-function mouse model for conditional inhibition of IKK2 in pancreatic β cells. (A) Transgenic approach for Dox regulated expression of IKK2 DN in pancreatic β cells. The tTA protein is expressed under the control of the endogenous Pdx1 promoter and can bind to the bid [file supplementary_figure_1.pdf]

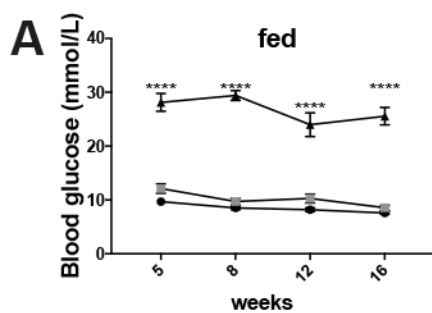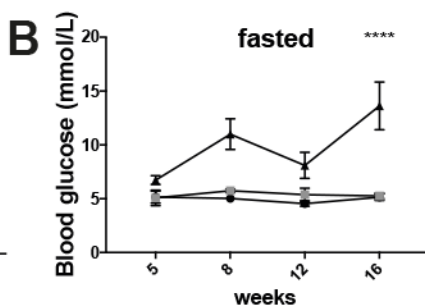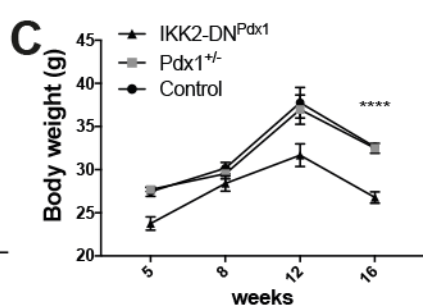

Supplement: Supplementary Figure 2: Analysis of disease progression in IKK2 DNPdx1 mice. (A) Fed and (B) fasted blood glucose levels and (C) body weight of 5 (n=4 8/group), 8 (n=5 9/group), 12 (n=6 9/group) and 16 (n=12 17/group) week old IKK2 DNPdx1 mice and control littermates are shown. Fed blood glucose lev [file supplementary_figure_2.pdf]

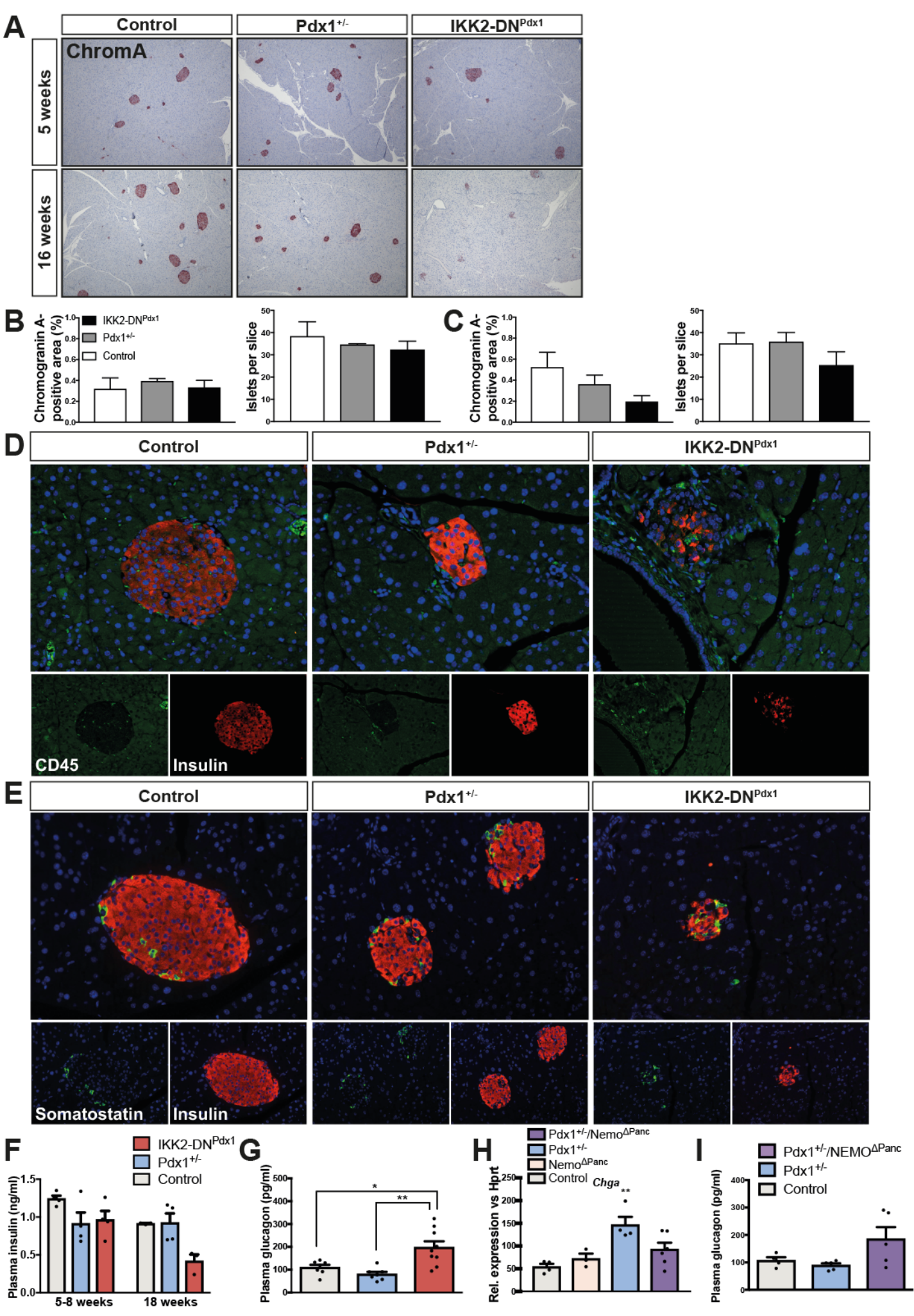

Supplement: Supplementary Figure 3: Islet morphology in IKK2 DNPdx1 mice. (A) Representative pictures of chromogranin A stained pancreata of 5 and 16 week old animals. (B, C) Quantification of total chromogranin A-positive area (% of total pancreatic area/slice, left panel) and islet number (right panel) was as [file supplementary_figure_3.pdf]
