# Supplementary material for: Elevated β-cell stress levels promote severe diabetes development in mice with MODY4
Source: J Endocrinol. 2019 Nov 4;244(2):323–37. doi: 10.1530/JOE-19-0208 (PMC6933809; doi:10.1530/JOE-19-0208)
Supplement: Supplementary Figure 4: Cellular Stress is increased in islets of IKK2 DNPdx1 mice. (A) Immunoblot of pancreatic islet extracts from 18 week old mice. (B-D) Quantification of protein expression levels for Atf6α, Hmox1 and Hsp27 (n=6/group for control and Pdx1+/-, n=7 for IKK2-DNPdx1 animals). The AU [file supplementary_figure_4.pdf]

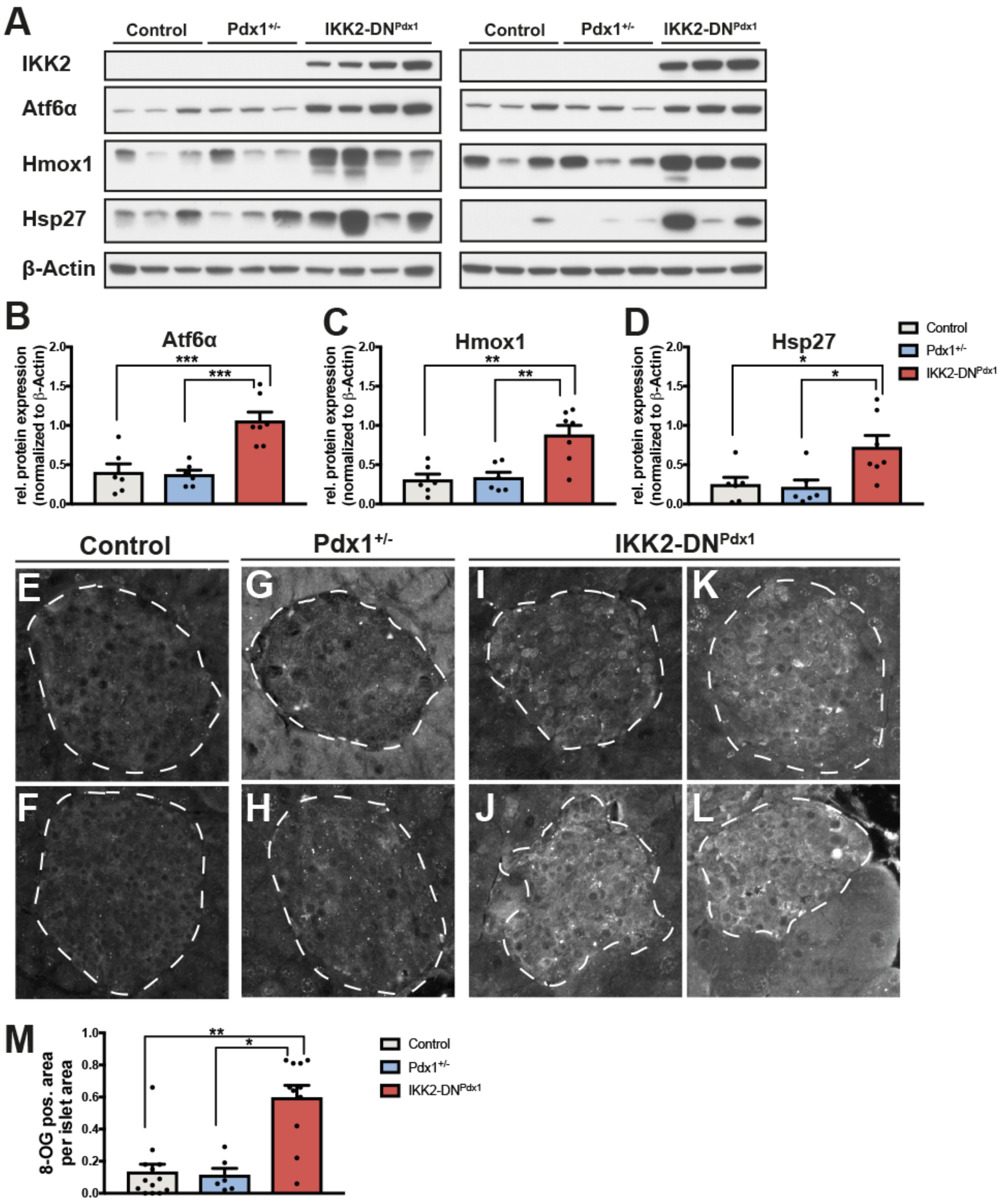
A
Control
Pdx1+/-
IKK2-DNPdx1
IKK2
Atf6α
Hmox1
Hsp27
β-Actin
B
Atf6α
rel. protein expression (normalized to β-Actin)
***
C
Hmox1
**
D
Hsp27
*
Control
Pdx1+/-
IKK2-DNPdx1
E
F
G
H
I
J
K
L
M
8-OG pos. area per islet area
**
*
