# Supplementary material for: Elevated β-cell stress levels promote severe diabetes development in mice with MODY4
Source: J Endocrinol. 2019 Nov 4;244(2):323–37. doi: 10.1530/JOE-19-0208 (PMC6933809; doi:10.1530/JOE-19-0208)
Supplement: Supplementary Table 1: Composition of mouse standard chow diet. [file supplementary_table_1.pdf]

Supplementary Table 1: Composition of mouse standard chow diet.

**Crude Nutrients**	**%**
Crude protein	22.0
Crude fat	4.5
Crude fiber	3.9
Crude ash	6.7
Calcium	1.0
Phosphorus	0.7
