# Supplementary material for: Elevated β-cell stress levels promote severe diabetes development in mice with MODY4
Source: J Endocrinol. 2019 Nov 4;244(2):323–37. doi: 10.1530/JOE-19-0208 (PMC6933809; doi:10.1530/JOE-19-0208)
Supplement: Supplementary Table 2: Antibodies used for western immunoblotting, immunohistochemistry and immunofluorescence staining. [file supplementary_table_2.pdf]

Supplementary Table 2: **Antibodies used for western immunoblotting, immunohistochemistry and immunofluorescence staining.**

Antibody	Company	Cat. Number
ATF6	Bioss	Bs-1634R
CD45	BD Bioscience	550539
Chromogranin A	Abcam	ab15160
ERK 2	Santa Cruz	sc-154
Glucagon	Cell Signaling	#2760
HO1	Enzo Life Science	SPA-895
HSP27	Santa Cruz	sc-13132
IKK2	Abcam	ab32135
IKKα/β	Santa Cruz	sc-7607
IKK2	Santa Cruz	sc-7329
Insulin	Abcam	ab7842
Ki-67	Thermo Scientific	RM-9106-S1
NEMO	BD Bioscience	611306
RelA/p65	Santa Cruz	sc-372
Somatostain	Santa Cruz	sc-13099
8-Oxoguanine	Merk	MAB3560
