# Supplementary material for: Elevated β-cell stress levels promote severe diabetes development in mice with MODY4
Source: J Endocrinol. 2019 Nov 4;244(2):323–37. doi: 10.1530/JOE-19-0208 (PMC6933809; doi:10.1530/JOE-19-0208)
Supplement: Supplementary Table 3: Pathway analysis of deregulated genes identified by microarray-based gene expression profiling of IKK2 DNPdx1 mice at early and later disease phases. Microarray analysis was performed with whole islet mRNA of 5 week and 18 week old mice (n=4 6). Data were analyzed with the Gen [file supplementary_table_3.pdf]

<u>Supplementary Table 3:</u> Pathway analysis of deregulated genes identified by microarray-based gene expression profiling of IKK2-DN^{Pdx1} mice at early and later disease phases.

Microarray analysis was performed with whole islet mRNA of 5 week and 18 week old mice (n=4-6). Data were analyzed with the Genesifter software using ANOVA analysis followed by Benjamini and Hochberg post-test. Pathway analysis was performed with the REACTOME software taking deregulated genes into account with a log2 fold change ≥ 1.5 compared to Pdx1$^{+/-}$ animals at 5 or 18 weeks of age. Selected pathways associated with β-cell dysfunction and cellular stress are depicted. qRT-PCR validated genes are underlined. Genes associated with T2D loci as identified by GWAS Catalog and FUMA are indicated.

Gene ID	Fold Change 5 weeks	Fold Change 18 weeks	Gene Title	ANOVA	Pdx1$^{+/-}$ 5 weeks mean	IKK2-DN^{Pdx1} 5 weeks mean	Pdx1$^{+/-}$ 18 weeks mean	IKK2-DN^{Pdx1} 18 weeks mean	Pdx1$^{+/-}$ 5 weeks SEM	IKK2-DN^{Pdx1} 5 weeks SEM	Pdx1$^{+/-}$ 18 weeks SEM	IKK2-DN^{Pdx1} 18 weeks SEM	Target Common Name	GWAS reported Genes	FUMA mapped loci 500kb	GwasPval	GWAS_SNPs
**Hormones:**																	
**<u>Ins1</u>**	**0,80**	**0,39**	Insulin I (Ins1), mRNA	0.000010	12.935300	12.609900	13.633200	12.281500	0.092451	0.135685	0.045221	0.234614	INS	GWAS mapped loci	FUMA mapped loci 500kb	0,00000003	rs3842770
**<u>Gcg</u>**	**1,11**	**0,62**	Glucagon, mRNA (cDNA clone MGC:14058 IMAGE:4218718)	0.042264	13.025000	13.171100	12.897500	12.211600	0.127908	0.044055	0.173974	0.459072	GCG				
**Sst**	**1,06**	**0,42**	Somatostatin, mRNA (cDNA clone MGC:18606 IMAGE:4218815)	0.000607	12.146700	12.232000	12.465500	11.220200	0.228207	0.120050	0.150315	0.184821	SST		FUMA mapped loci 500kb	4,38E-13	rs11923275; rs6808574
**<u>Cck</u>**	**7,37**	**2,04**	Cholecystokinin, mRNA (cDNA clone MGC:41001 IMAGE:1400830)	0.000032	6.054430	8.936330	4.261970	5.291350	0.559817	0.680711	0.268889	0.450194	CCK				
**β-cell function & insulin secretion:**																	
**<u>Slc2a2</u>**	**0,60**	**0,16**	Solute carrier family 2 (facilitated glucose transporter), member 2, mRNA (cDNA clone MGC:25413 IMAGE:4236331)	0.000045	9.402980	8.674740	9.660770	6.973210	0.405893	0.269465	0.166847	0.348744	SLC2A2	GWAS mapped loci	FUMA mapped loci 500kb	2,25E-08	rs11925227
**<u>Ucn3</u>**	**0,31**	**0,12**	Urocortin 3 (Ucn3), mRNA	0.000142	8.948490	7.241780	8.285590	5.272810	0.503670	0.549963	0.153211	0.172265	UCN3				
**<u>Insrr</u>**	**0,46**	**0,12**	Insulin receptor-related receptor (Insrr), mRNA	0.000038	9.327500	8.198930	8.823180	5.780010	0.477841	0.442319	0.195873	0.127152	INSRR				
**<u>G6pc2</u>**	**0,50**	**0,11**	Glucose-6-phosphatase, catalytic, 2 (G6pc2), mRNA	0.000045	11.301300	10.301300	11.495000	8.295200	0.416449	0.425081	0.134027	0.337054	G6PC2	GWAS mapped loci	FUMA mapped loci 500kb	3E-11	rs3755157
**<u>Slc30a8</u>**	**0,41**	**0,09**	Solute carrier family 30 (zinc transporter), member 8, mRNA (cDNA clone MGC:144753 IMAGE:40105622)	0.000015	10.417200	9.128540	10.209400	6.712450	0.488778	0.336612	0.162524	0.513803	SLC30A8	GWAS mapped loci	FUMA mapped loci 500kb	2,32E-52	rs3802177
**<u>Chga</u>**	**0,91**	**0,23**	Chromogranin A, mRNA (cDNA clone MGC:36117 IMAGE:4990941)	0.000005	12.897400	12.757200	13.065400	10.930500	0.131389	0.130151	0.140399	0.371083	CHGA				

**Iapp**	**1,03**	**0,45**	Islet amyloid polypeptide, mRNA (cDNA clone MGC:41171 IMAGE:1379049)	0.000205	12.969900	13.007600	13.127600	11.977400	0.161000	0.071759	0.095921	0.269641	IAPP				
**Glp1r**	**0,75**	**0,35**	Glucagon-like peptide 1 receptor (Glp1r), mRNA	0.001635	10.960900	10.541900	10.382700	8.861070	0.360040	0.281484	0.221023	0.341541	GLP1R				
**Pcsk1n**	**1,10**	**0,34**	Proprotein convertase subtilisin/kexin type 1 inhibitor, mRNA (cDNA clone MGC:19107 IMAGE:4207854)	0.000000	10.788300	10.930000	9.273260	7.710720	0.278812	0.190363	0.250798	0.102475	PCSK1N				
**Isl1**	**0,89**	**0,33**	ISL1 transcription factor, LIM/homeodomain (Isl1), mRNA	0.001412	8.689460	8.521260	8.809150	7.230150	0.365902	0.214252	0.178193	0.194132	ISL1				
**Pcsk1**	**0,91**	**0,25**	Proprotein convertase subtilisin/kexin type 1 (Pcsk1), mRNA	0.000003	10.496600	10.355800	8.789750	6.761620	0.524178	0.311757	0.194194	0.290715	PCSK1	GWAS mapped loci	FUMA mapped loci 500kb	5E-15	rs6235
**Pcsk2**	**1,21**	**0,36**	Strain ILS Kex2-like protein	0.000111	12.000900	12.279300	11.715600	10.230500	0.316755	0.152418	0.201443	0.310164	PCSK2				
**Cpe**	**1,14**	**0,29**	Carboxypeptidase E, mRNA (cDNA clone MGC:7101 IMAGE:3157558)	0.000218	11.317900	11.502200	11.137900	9.338250	0.412620	0.186878	0.218691	0.314438	CPE				
**Prlr**	**0,69**	**0,19**	Prolactin receptor, mRNA (cDNA clone IMAGE:5337438)	0.000000	9.119710	8.587370	6.805270	4.408240	0.528895	0.324397	0.241101	0.263615	PRLR				
**Gna15**	**0,79**	**2,43**	Guanine nucleotide binding protein, alpha 15, mRNA (cDNA clone MGC:18931 IMAGE:3967497)	0.000414	6.616890	6.280250	5.426680	6.710590	0.350061	0.072761	0.147990	0.176929	GNA15				
**Plcb2**	**0,92**	**1,86**	Phospholipase C, beta 2, mRNA (cDNA clone IMAGE:4020152)	0.000307	6.258460	6.144490	5.191610	6.087700	0.235209	0.112982	0.120212	0.182722	PLCB2				
**Gng2**	**0,94**	**1,86**	Guanine nucleotide binding protein (G protein), gamma 2, mRNA (cDNA clone MGC:36070 IMAGE:5150203)	0.000349	7.921000	7.838810	6.925520	7.819650	0.225590	0.053846	0.152974	0.174185	GNG2				
**Marcks**	**0,90**	**1,73**	Myristoylated alanine rich protein kinase C substrate (Marcks), mRNA	0.000000	10.256100	10.106400	6.997320	7.788180	0.208438	0.105453	0.111690	0.199389	MARCKS				
**Cacna1a**	**0,89**	**0,65**	Cacna1a mRNA for CaV2.1, complete cds, clone:MPI	0.000099	8.924290	8.748420	7.918440	7.291550	0.281872	0.158489	0.186949	0.116513	CACNA1A				
**Gnas**	**1,17**	**0,65**	GNAS (guanine nucleotide binding protein, alpha stimulating) complex locus, mRNA (cDNA clone IMAGE:4013293)	0.000021	9.338800	9.565330	8.523080	7.892600	0.111580	0.037949	0.200712	0.293573	GNAS				
**Prkca**	**1,00**	**0,64**	Protein kinase C, alpha (Prkca), mRNA	0.000000	8.345310	8.341270	6.571090	5.927320	0.263580	0.095545	0.141771	0.059168	PRKCA				
**Ahcyl2**	**0,98**	**0,63**	S-adenosylhomocysteine hydrolase-like 2 (Ahcyl2), mRNA	0.000000	9.643730	9.621360	7.783490	7.112460	0.258965	0.130012	0.081977	0.185922	AHCYL2				
**Adcy6**	**1,05**	**0,62**	Adenylate cyclase 6 (Adcy6), mRNA	0.000000	8.626340	8.690070	7.370690	6.677760	0.139093	0.041705	0.146464	0.067002	ADCY6				
**Itpr3**	**0,96**	**0,61**	Inositol 1,4,5-triphosphate receptor 3, mRNA (cDNA clone IMAGE:5345968)	0.000000	8.884030	8.821990	7.887670	7.176580	0.224765	0.061106	0.141708	0.037712	ITPR3				
**Gng12**	**0,98**	**0,61**	Guanine nucleotide binding protein (G protein), gamma 12 (Gng12), mRNA	0.000000	11.711100	11.674600	9.604420	8.890050	0.220396	0.103043	0.106060	0.143392	GNG12				
**Ahcyl1**	**0,90**	**0,61**	S-adenosylhomocysteine hydrolase-like 1 (Ahcyl1), mRNA	0.000000	9.896430	9.750690	9.176980	8.459940	0.147094	0.064991	0.047968	0.065708	AHCYL1				
**Prkar1a**	**1,16**	**0,60**	Protein kinase, cAMP dependent regulatory, type I, alpha, mRNA (cDNA clone MGC:11864 IMAGE:3597765)	0.000000	10.655900	10.867600	9.220490	8.472310	0.153872	0.087670	0.160660	0.069926	PRKAR1A				
**Cacnb3**	**1,05**	**0,58**	Strain ILS calcium channel beta 3 subunit	0.000000	8.873960	8.950720	7.147280	6.366890	0.203571	0.069062	0.147165	0.222999	CACNB3				
**Adra2a**	**0,96**	**0,57**	Adrenergic receptor, alpha 2a (Adra2a), mRNA	0.000011	7.925370	7.873770	6.538200	5.735320	0.332537	0.192321	0.221749	0.133344	ADRA2A				
**Snap25**	**1,54**	**0,54**	Strain ILS synaptosomal associated protein 25	0.000053	9.764850	10.389100	8.978030	8.100440	0.456535	0.166193	0.117668	0.307593	SNAP25				

**Kcnb1**	**0,94**	**0,54**	Potassium voltage gated channel, Shab-related subfamily, member 1, mRNA (cDNA clone MGC:25500 IMAGE:4507847)	0.000026	7.990530	7.899100	7.111060	6.231020	0.295356	0.179705	0.130817	0.109352	KCNB1			
**Vamp2**	**0,96**	**0,54**	Vesicle-associated membrane protein 2, mRNA (cDNA clone MGC:49500 IMAGE:3156993)	0.000000	10.040100	9.979320	8.950450	8.050330	0.157106	0.094287	0.159163	0.057077	VAMP2			
**Cacnb2**	**0,99**	**0,53**	L-type calcium channel beta 2a subunit isoform mRNA, 5' and 3' flanks	0.000159	8.004090	7.991250	7.360960	6.447440	0.329937	0.181474	0.131902	0.090643	CACNB2			
**Prkar1b**	**0,98**	**0,52**	Protein kinase, cAMP dependent regulatory, type I beta, mRNA (cDNA clone MGC:18526 IMAGE:3674751)	0.000012	8.776610	8.752700	7.704800	6.771340	0.385949	0.190041	0.118800	0.020984	PRKAR1B			
**Gng4**	**1,12**	**0,51**	Guanine nucleotide binding protein (G protein), gamma 4, mRNA (cDNA clone MGC:25282 IMAGE:4502719)	0.000000	8.918890	9.080040	7.296410	6.324220	0.384672	0.191415	0.165290	0.118040	GNG4			
**Syt5**	**1,31**	**0,49**	Synaptotagmin V (Syt5), mRNA	0.000002	9.232540	9.627250	7.258910	6.225980	0.509737	0.219143	0.261914	0.277481	SYT5			
**Prkacb**	**0,90**	**0,49**	Protein kinase, cAMP dependent, catalytic, beta (Prkacb), mRNA	0.000001	10.733400	10.586000	9.524700	8.489970	0.301900	0.166810	0.100093	0.120598	PRKACB			
**Stxbp1**	**0,99**	**0,47**	Syntaxin binding protein 1, mRNA (cDNA clone MGC:30503 IMAGE:4480978)	0.000002	9.268520	9.258790	8.473600	7.381120	0.266383	0.136654	0.154139	0.033888	STXBP1			
**Itpr1**	**0,91**	**0,44**	Inositol 1,4,5-triphosphate receptor 1, mRNA (cDNA clone IMAGE:3499330)	0.000000	8.708290	8.565760	7.844620	6.673780	0.151671	0.087697	0.173034	0.116592	ITPR1			
**Ffar1**	**0,90**	**0,44**	Free fatty acid receptor 1, mRNA (cDNA clone MGC:169723 IMAGE:8861118)	0.001365	7.954930	7.807580	6.781170	5.594710	0.525752	0.392685	0.230036	0.164220	FFAR1			
**Rapgef4**	**0,72**	**0,26**	CAMP-GEFII	0.000029	9.592470	9.119990	8.335590	6.374860	0.498591	0.323261	0.210593	0.171132	RAPGEF4	GWAS mapped loci	FUMA mapped loci 500kb	7E-11	rs733331
**Developmental regulation:**																
**Mafb**	**0,85**	**2,55**	V-maf musculoaponeurotic fibrosarcoma oncogene family, protein B (avian) (Mafb), mRNA	0.000027	9.341420	9.101430	7.089220	8.442390	0.392049	0.100328	0.277448	0.241685	MAFB			
**Slc38a5**	**1,19**	**2,36**	Solute carrier family 38, member 5, mRNA (cDNA clone MGC:173142 IMAGE:40057282)	0.000155	9.466380	9.721450	8.098240	9.335310	0.261670	0.085061	0.228492	0.311089	SLC38A5			
**Rbpj**	**0,97**	**2,14**	Recombining binding protein suppressor of hairless (Drosophila), mRNA (cDNA clone IMAGE:3983555)	0.022130	5.299970	5.258510	4.505250	5.600500	0.378329	0.088263	0.198560	0.315392	RBPJ			
**Ptf1a**	**1,05**	**1,87**	Pancreas specific transcription factor, 1a (Ptf1a), mRNA	0.000061	7.216800	7.286560	5.729860	6.632120	0.405557	0.077670	0.143004	0.162193	PTF1A			
**Onecut1**	**1,51**	**0,76**	One cut domain, family member 1, mRNA (cDNA clone MGC:36136 IMAGE:5101697)	0.000106	6.095600	6.691730	5.839860	5.449700	0.143695	0.170685	0.126959	0.029120	ONECUT1			
**Notch1**	**0,93**	**0,64**	Notch 1 protein	0.000000	9.164140	9.056510	8.017800	7.365360	0.082877	0.042922	0.112064	0.133195	NOTCH1			
**Hes1**	**1,66**	**0,62**	Hairy and enhancer of split 1 (Drosophila), mRNA (cDNA clone MGC:25356 IMAGE:4505427)	0.000005	8.208930	8.938770	7.175850	6.491010	0.207353	0.104065	0.318234	0.136615	HES1			
**Tshz1**	**0,90**	**0,62**	Teashirt zinc finger family member 1, mRNA (cDNA clone IMAGE:5372548)	0.000000	8.879010	8.729690	7.577180	6.889070	0.110216	0.070419	0.180520	0.055856	TSHZ1			
**Foxa2**	**1,00**	**0,53**	Forkhead box A2 (Foxa2), mRNA	0.020670	7.555920	7.551430	7.659690	6.732840	0.280845	0.181109	0.173498	0.109534	FOXA2	GWAS mapped loci	FUMA mapped loci 500kb	3,34E-08	rs6515236

**Sox9**	**1,67**	**0,48**	SRY-box containing gene 9, mRNA (cDNA clone MGC:38112 IMAGE:5320371)	0.000138	8.252290	8.988990	7.500250	6.447800	0.223938	0.151806	0.418646	0.168948	SOX9				
**Insm1**	**1,16**	**0,47**	Insulinoma-associated 1 (Insm1), mRNA	0.000008	8.681270	8.900490	7.690120	6.586290	0.318881	0.203486	0.150925	0.244958	INSM1				
**Pdx1**	**1,04**	**0,45**	Pancreatic and duodenal homeobox 1 (Pdx1), mRNA	0.001021	7.658920	7.714760	7.004930	5.844800	0.444592	0.263094	0.193294	0.158038	PDX1	GWAS mapped loci	FUMA mapped loci 500kb	9E-10	rs2293941
**Hnf4g**	**1,08**	**0,36**	Hepatocyte nuclear factor 4, gamma (Hnf4g), mRNA	0.028896	5.572780	5.684750	5.938580	4.447490	0.423892	0.180579	0.397356	0.082212	HNF4G				
**Nkx6-1**	**0,81**	**0,30**	NK6 homeobox 1 (Nkx6-1), mRNA	0.000803	8.771480	8.463420	8.372740	6.657960	0.425213	0.296655	0.205010	0.170352	NKX6-1				
**Nkx2-2**	**1,25**	**0,27**	NK2 transcription factor related, locus 2 (Drosophila) (Nkx2-2), transcript variant 1, mRNA	0.000144	8.353560	8.680090	8.252160	6.369640	0.480086	0.230083	0.210317	0.111954	NKX2-2				
**Abcc8**	**1,06**	**0,22**	ATP-binding cassette, sub-family C (CFTR/MRP), member 8, mRNA (cDNA clone MGC:176373 IMAGE:9056024)	0.000032	10.545200	10.626500	9.879690	7.726700	0.467979	0.227512	0.247814	0.341728	ABCC8		FUMA mapped loci 500kb	2,09E-20	rs5215
**Hnf1b**	**1,65**	**0,22**	HNF1 homeobox B, mRNA (cDNA clone MGC:32464 IMAGE:5044299)	0.000307	7.946230	8.667070	8.190440	6.034210	0.133207	0.157415	0.454234	0.325897	HNF1B	GWAS mapped loci	FUMA mapped loci 500kb	8,98E-22	rs11651755
**Gjd2**	**0,75**	**0,22**	Gap junction protein, delta 2 (Gjd2), mRNA	0.000001	9.386840	8.973280	7.145350	4.963210	0.555148	0.314328	0.251721	0.294444	GJD2				
**Pax6**	**1,00**	**0,20**	Paired box gene 6, mRNA (cDNA clone MGC:46875 IMAGE:4504106)	0.000036	9.042760	9.042410	8.664820	6.371050	0.454232	0.276575	0.215079	0.220653	PAX6				
**Gcgr**	**1,88**	**0,96**	Glucagon receptor, mRNA (cDNA clone MGC:30235 IMAGE:5137340)	0.000001	6.662260	7.574400	5.797130	5.735850	0.245680	0.156204	0.156519	0.107246	GCGR				
**Neurod1**	**0,95**	**0,16**	Neurogenic differentiation 1, mRNA (cDNA clone MGC:25680 IMAGE:4511370)	0.000007	8.977070	8.910250	8.007020	5.337120	0.480854	0.286223	0.331320	0.233395	NEUROD1				
**Cell-cycle check points:**																	
**Zfp385a**	**1,01**	**1,80**	Zinc finger protein 385A, mRNA (cDNA clone MGC:28982 IMAGE:4504518)	0.000182	8.225340	8.233850	6.778110	7.626070	0.404006	0.063311	0.198273	0.098858	ZNF385A				
**Aurkb**	**1,20**	**1,62**	Aurora kinase B, mRNA (cDNA clone MGC:5803 IMAGE:3501444)	0.000000	7.121480	7.388800	5.663670	6.361870	0.041914	0.147307	0.111906	0.038856	AURKB				
**Dna2**	**1,16**	**1,52**	DNA replication helicase 2 homolog (yeast), mRNA (cDNA clone IMAGE:5376010)	0.000013	6.076620	6.287110	4.907880	5.509990	0.095322	0.196012	0.081539	0.161593	DNA2				
**Mcm3**	**1,54**	**1,22**	Minichromosome maintenance deficient 3 (S. cerevisiae), mRNA (cDNA clone MGC:30531 IMAGE:5007886)	0.000060	6.153120	6.773190	5.233060	5.524560	0.136031	0.280192	0.084846	0.045748	MCM3				
**Cdkn1a**	**1,60**	**1,12**	Cyclin-dependent kinase inhibitor 1A (P21) (Cdkn1a), transcript variant 1, mRNA	0.000000	9.381900	10.059100	6.961900	7.120770	0.299912	0.162709	0.306922	0.111083	CDKN1A				
**Sfn**	**1,54**	**0,79**	Stratifin (Sfn), mRNA	0.006600	6.428740	7.054290	6.648780	6.312970	0.056885	0.128438	0.146548	0.157488	SFN				
**Ppp2r5c**	**0,93**	**0,66**	Protein phosphatase 2, regulatory subunit B (B56), gamma isoform (Ppp2r5c), transcript variant 1, mRNA	0.000000	9.428900	9.328440	7.710420	7.113050	0.262688	0.138187	0.150164	0.125366	PPP2R5C				
**Rad9b**	**1,15**	**0,65**	RAD9 homolog B (S. cerevisiae) (Rad9b), mRNA	0.000304	5.989350	6.186340	5.473690	4.849010	0.259758	0.182608	0.114627	0.095381	RAD9B				
**Myt1**	**0,92**	**0,64**	Neural zinc finger protein NZF-2b	0.000435	6.973390	6.847050	6.073030	5.431500	0.412282	0.176126	0.113073	0.088310	MYT1				
**Aim1**	**1,45**	**0,62**	Absent in melanoma 1, mRNA (cDNA clone IMAGE:5370522)	0.000278	7.205740	7.742610	6.828980	6.148650	0.069399	0.098470	0.277780	0.168495	AIM1				

**Slc22a5**	**1,00**	**0,62**	Solute carrier family 22 (organic cation transporter), member 5, mRNA (cDNA clone MGC:36043 IMAGE:4981427)	0.000000	7.514790	7.509720	5.931440	5.230470	0.054412	0.024052	0.160546	0.205320	SLC22A5		
**Dynll2**	**0,87**	**0,55**	Dynein light chain LC8-type 2 (Dynll2), mRNA	0.000000	9.794220	9.598600	8.229340	7.368690	0.184719	0.113655	0.167963	0.095317	DYNLL2	FUMA mapped loci 500kb	2,46E-08	rs302864
**Cds1**	**0,85**	**0,47**	CDP-diacylglycerol synthase 1, mRNA (cDNA clone MGC:62814 IMAGE:6491596)	0.000134	7.496200	7.253740	6.420730	5.335490	0.394981	0.284077	0.142083	0.065322	CDS1		
**Spc25**	**0,81**	**0,32**	SPC25, NDC80 kinetochore complex component, homolog (S. cerevisiae), mRNA (cDNA clone MGC:40860 IMAGE:5369405)	0.000217	8.338870	8.026210	7.310330	5.669630	0.464188	0.379185	0.171683	0.091149	SPC25		
**Ccna2**	**1,24**	**1,59**	Cyclin A2 (Ccna2), mRNA	0.000002	6.929550	7.240520	5.435300	6.107060	0.139274	0.222681	0.107752	0.106950	CCNA2		
**Cellular senescnce:**															
**Trf**	**0,82**	**3,33**	Transferrin (Trf), mRNA	0.000007	11.017500	10.739300	8.377500	10.112600	0.377851	0.084392	0.336772	0.173203	TF		
**Hist1h2ab**	**1,44**	**1,68**	Histone cluster 1, H2ab, mRNA (cDNA clone MGC:151487 IMAGE:40126429)	0.000558	4.621340	5.146670	4.035760	4.784080	0.120629	0.158505	0.110115	0.244499	NA		
**Hist1h3f**	**1,23**	**1,52**	Histone cluster 1, H3f (Hist1h3f), mRNA	0.000000	10.816300	11.113800	9.350730	9.959140	0.075939	0.115367	0.136868	0.161034	NA		
**Hist1h3g**	**1,23**	**1,51**	Histone cluster 1, H3g (Hist1h3g), mRNA	0.000000	10.737400	11.038500	9.369230	9.963770	0.075239	0.113725	0.123305	0.148409	HIST1H3A		
**Cebpb**	**1,05**	**1,53**	CCAAT/enhancer binding protein (C/EBP), beta (Cebpb), mRNA	0.001887	8.160280	8.226890	7.183210	7.794890	0.263156	0.167137	0.160168	0.127950	CEBPB		
**Hist1h3a**	**1,23**	**1,51**	Histone cluster 1, H3a (Hist1h3a), mRNA	0.000000	10.756900	11.055000	9.330040	9.922480	0.074571	0.110409	0.117250	0.154315	HIST1H3E		
**Cdk6**	**1,55**	**1,22**	Cyclin-dependent kinase 6 (Cdk6), mRNA	0.000000	8.015790	8.647130	6.789600	7.072530	0.259338	0.131818	0.128403	0.077069	CDK6		
**Jun**	**0,98**	**0,66**	Jun oncogene, mRNA (cDNA clone MGC:11557 IMAGE:3156091)	0.000001	12.045700	12.021100	10.038000	9.435860	0.101875	0.058095	0.375502	0.082882	JUN		
**Terf2**	**0,94**	**0,65**	Telomeric repeat binding factor 2 (Terf2), transcript variant 1, mRNA	0.000003	7.720340	7.626610	6.922550	6.304590	0.205140	0.101913	0.112490	0.078572	TERF2	FUMA mapped loci 500kb	3,88E-09	rs244415
**Ets2**	**1,15**	**0,65**	E26 avian leukemia oncogene 2, 3' domain, mRNA (cDNA clone MGC:7483 IMAGE:3490813)	0.000000	9.956790	10.156600	8.373550	7.741360	0.128399	0.047495	0.158151	0.082981	ETS2		
**Phc3**	**1,00**	**0,64**	Polyhomeotic-like 3 (Drosophila) (Phc3), mRNA	0.000007	8.133830	8.134720	7.500740	6.854020	0.064340	0.055335	0.183388	0.075608	PHC3		
**Tnrc6a**	**0,94**	**0,63**	Trinucleotide repeat containing 6a (Tnrc6a), mRNA	0.000003	8.629460	8.541540	7.933570	7.273390	0.156886	0.061965	0.131229	0.131307	TNRC6A		
**Kdm6b**	**1,13**	**0,59**	Jumonji domain containing 3, mRNA (cDNA clone IMAGE:1378940)	0.000000	8.969850	9.147230	7.790510	7.027080	0.199479	0.160154	0.165510	0.089511	KDM6B		
**Rps6ka2**	**0,89**	**0,41**	Ribosomal protein S6 kinase, polypeptide 2, mRNA (cDNA clone IMAGE:4505898)	0.000087	8.912220	8.735990	7.606080	6.307080	0.435044	0.306539	0.251871	0.076160	RPS6KA2		
**Mapk10**	**1,56**	**0,40**	Mitogen-activated protein kinase 10 (Mapk10), transcript variant 1, mRNA	0.000341	8.923530	9.567690	8.457430	7.141550	0.519492	0.245782	0.194409	0.263973	MAPK10		
**Cdh1**	**1,49**	**0,38**	Cadherin 1 (Cdh1), mRNA	0.000029	9.997530	10.574500	9.522610	8.111070	0.250534	0.144179	0.295304	0.243811	CDH1		
**Il1a**	**2,68**	**1,09**	Interleukin 1 alpha, mRNA (cDNA clone MGC:5780 IMAGE:3599550)	0.000005	6.311430	7.733240	3.782730	3.907920	0.795426	0.456261	0.077773	0.111804	IL1A		
**Il6**	**2,85**	**1,02**	Interleukin 6 (Il6), mRNA	0.000000	7.148690	8.660640	3.736380	3.763520	0.717254	0.435888	0.235742	0.157038	IL6		
**ROS detoxification:**															

**Cybb**	**1,23**	**4,69**	Cytochrome b-245, beta polypeptide (Cybb), mRNA	0.000036	9.507030	9.808850	6.911470	9.141840	0.651979	0.179043	0.314211	0.220786	CYBB			
**Hmox1**	**1,37**	**3,19**	Heme oxygenase (decycling) 1, mRNA (cDNA clone MGC:18463 IMAGE:4235374)	0.005352	8.515350	8.968380	6.603250	8.277350	0.719985	0.184710	0.536375	0.127959	HMOX1			
**Ncf1**	**1,07**	**2,87**	Neutrophil cytosolic factor 1 (Ncf1), mRNA	0.018635	7.851200	7.951700	6.820280	8.341740	0.607497	0.169566	0.254675	0.264861	NCF1			
**Ncf2**	**1,18**	**2,75**	Neutrophil cytosolic factor 2, mRNA (cDNA clone MGC:5795 IMAGE:3592460)	0.001297	7.677000	7.912090	6.248480	7.705550	0.557638	0.148413	0.242875	0.111479	NCF2			
**Ctse**	**1,45**	**2,54**	Cathepsin E, mRNA (cDNA clone MGC:5924 IMAGE:3601519)	0.022604	6.549260	7.086130	5.767430	7.110040	0.429092	0.407559	0.132701	0.238637	CTSE			
**Aqp8**	**1,21**	**2,14**	Aquaporin 8, mRNA (cDNA clone MGC:13894 IMAGE:4224850)	0.000007	6.881700	7.160520	4.931530	6.031420	0.419403	0.108170	0.179250	0.257656	AQP8			
**Ncf4**	**0,92**	**2,07**	Neutrophil cytosolic factor 4, mRNA (cDNA clone MGC:36154 IMAGE:5321182)	0.008628	7.009190	6.893810	5.960440	7.011840	0.421281	0.170086	0.153915	0.184797	NCF4			
**Cyba**	**1,20**	**1,70**	Cytochrome b-245, alpha polypeptide (Cyba), mRNA	0.000001	9.731760	9.997140	7.635130	8.401580	0.444760	0.157665	0.111974	0.196868	CYBA			
**Gpx1**	**0,97**	**1,64**	Glutathione peroxidase 1 (Gpx1), mRNA	0.000000	10.518900	10.482300	9.129350	9.847320	0.202725	0.054417	0.066015	0.108565	GPX1	GWAS mapped loci	FUMA mapped loci 500kb	8E-10	rs9818758
**Nox4**	**1,16**	**1,55**	Superoxide-generating NADPH oxidase 4 (Nox4)	0.000002	6.720050	6.929510	4.968590	5.599640	0.377197	0.136018	0.052659	0.204796	NOX4			
**Gpx2**	**1,65**	**0,67**	Glutathione peroxidase 2 (Gpx2), mRNA	0.000000	7.385090	8.107020	6.066030	5.483930	0.254973	0.108797	0.069585	0.146042	GPX2			
**Unfolded Protein Response (UPR):**																
**Edem1**	**1,01**	**1,63**	ER degradation enhancer, mannosidase alpha-like 1, mRNA (cDNA clone MGC:25513 IMAGE:2654113)	0.000000	10.383600	10.391800	8.609310	9.309810	0.059665	0.043569	0.174857	0.245470	EDEM1			
**Wfs1**	**0,93**	**0,40**	Wolfram syndrome 1 homolog (human) (Wfs1), mRNA	0.000001	8.976030	8.866790	7.875280	6.565750	0.311009	0.150713	0.173786	0.125376	WFS1	GWAS mapped loci	FUMA mapped loci 500kb	5,52E-34	rs1801214
**Hyou1**	**1,03**	**0,55**	Hypoxia up-regulated 1, mRNA (cDNA clone MGC:30561 IMAGE:5151731)	0.000158	10.489300	10.531800	9.786270	8.928790	0.331110	0.095220	0.169383	0.236357	HYOU1			
**Gfpt1**	**0,78**	**0,43**	Glutamine fructose-6-phosphate transaminase 1, mRNA (cDNA clone MGC:19057 IMAGE:4191553)	0.000176	10.044100	9.678610	9.208400	7.999140	0.399574	0.164234	0.134571	0.276333	GFPT1			
**Nfyc**	**0,97**	**0,63**	Nuclear transcription factor-Y gamma, mRNA (cDNA clone MGC:28940 IMAGE:4009737)	0.000000	8.726590	8.682010	7.241090	6.585510	0.093305	0.041906	0.083603	0.046493	NFYC			
**Creb3l2**	**1,02**	**0,40**	CAMP responsive element binding protein 3-like 2, mRNA (cDNA clone MGC:170095 IMAGE:8861490)	0.000000	9.714010	9.736690	8.446130	7.109520	0.173413	0.097664	0.136731	0.113516	CREB3L2			
**Sec31a**	**0,98**	**0,61**	SEC31-like 1 (S. cerevisiae), mRNA (cDNA clone IMAGE:5125246)	0.000001	10.606900	10.572400	10.114500	9.400810	0.143823	0.036339	0.055666	0.171625	SEC31A		FUMA mapped loci 500kb	4,59E-10	rs993380
**Tspyl2**	**0,90**	**0,58**	CASK interacting nucleosome assembly protein	0.000001	8.936220	8.792150	7.858970	7.065430	0.181622	0.103143	0.175861	0.062709	TSPYL2			
**Atf3**	**1,19**	**0,40**	Activating transcription factor 3, mRNA (cDNA clone MGC:28295 IMAGE:4011514)	0.000224	11.936500	12.183500	10.266000	8.955370	0.229350	0.074112	0.637602	0.296013	ATF3			
**Dnajb9**	**0,97**	**0,36**	DnaJ (Hsp40) homolog, subfamily B, member 9, mRNA (cDNA clone IMAGE:5064823)	0.000000	11.034200	10.986900	9.514490	8.049630	0.339096	0.161983	0.146221	0.201063	DNAJB9			

**Serine proteases:**																
Gm10334 (EG436523)	**10,19**	**20,06**	Predicted gene, EG436523, mRNA (cDNA clone MGC:163657 IMAGE:40130303)	0.000001	8.225100	11.573500	6.552220	10.878200	0.613314	0.531554	0.194109	0.455847	NA			
Prss3	**7,88**	**11,21**	Protease, serine, 3 (Prss3), mRNA	0.000140	9.062050	12.040400	8.973420	12.460000	0.955812	0.389670	0.364159	0.352501	PRSS3	FUMA mapped loci 500kb	1,36E-09	rs1758632
**Il-10 signaling:**																
**Ccr2**	**1,58**	**4,60**	Chemokine (C-C motif) receptor 2 (Ccr2), mRNA	0.004673	6.001300	6.663920	5.274370	7.476320	0.456613	0.397248	0.292459	0.290044	CCR2			
**Ptafr**	**1,25**	**3,42**	Platelet activating factor receptor	0.000052	7.967310	8.288910	5.423820	7.196480	0.732016	0.140160	0.258916	0.233865	PTAFR			
**Il10ra**	**0,87**	**3,19**	Interleukin 10 receptor, alpha (Il10ra), mRNA	0.001784	7.548030	7.348450	5.709500	7.382300	0.658639	0.176617	0.218012	0.237045	IL10RA			
**Cd86**	**1,59**	**2,94**	CD86 antigen, mRNA (cDNA clone MGC:18471 IMAGE:4008635)	0.000120	7.333920	7.998620	5.227360	6.784410	0.714279	0.174654	0.220461	0.320807	CD86			
**Ccr1**	**1,41**	**2,51**	Chemokine (C-C motif) receptor 1, mRNA (cDNA clone MGC:18743 IMAGE:3992755)	0.011431	5.102240	5.598200	4.442360	5.771520	0.424427	0.263228	0.174888	0.263579	CCR1			
**Ccr5**	**1,33**	**2,25**	Chemokine (C-C motif) receptor 5 (Ccr5), mRNA	0.000214	6.260170	6.667390	4.555650	5.728750	0.353976	0.316212	0.233481	0.156672	CCR2			
**Ccl2**	**2,33**	**2,25**	Strain NOD/LtJ small inducible cytokine A2 precursor (Scya2)	0.000006	9.439410	10.659300	6.201150	7.368530	0.870538	0.273786	0.401285	0.139873	CCL2			
**Ccl3**	**2,10**	**2,00**	Chemokine (C-C motif) ligand 3 (Ccl3), mRNA	0.000008	10.484300	11.552200	6.839540	7.838850	0.943502	0.135717	0.471569	0.437472	CCL18			
**Ptgs2**	**1,58**	**1,81**	Prostaglandin-endoperoxide synthase 2 (Ptgs2), mRNA	0.000000	10.116400	10.774500	6.537510	7.392890	0.550643	0.243113	0.405411	0.205880	PTGS2			
**Tnfrsf1b**	**0,95**	**1,76**	Tumor necrosis factor receptor superfamily, member 1b (Tnfrsf1b), mRNA	0.000084	8.439520	8.364110	6.573420	7.392480	0.391224	0.123656	0.246278	0.228727	TNFRSF1B			
**Cd80**	**1,36**	**1,74**	Strain SJL/J B lymphocyte activation antigen CD80 precursor (Cd80)	0.016183	6.357600	6.800520	5.155440	5.950860	0.716467	0.275677	0.188943	0.177568	CD80			
**Ccl22**	**1,14**	**1,71**	Chemokine (C-C motif) ligand 22, mRNA (cDNA clone MGC:13812 IMAGE:4192393)	0.000035	5.781810	5.964750	4.399640	5.171160	0.328657	0.162015	0.070385	0.202386	CCL22			
**Il10rb**	**0,96**	**1,59**	C57BL/6 interleukin 10 receptor 2 precursor (Il10r2)	0.000193	9.414300	9.350060	8.292710	8.965500	0.323970	0.075726	0.089232	0.138291	IL10RB			
**Tnf**	**1,69**	**1,37**	Tumor necrosis factor (Tnf), mRNA	0.000000	8.043260	8.799940	5.354690	5.805500	0.627140	0.254928	0.097480	0.070817	TNF			
**Cxcl2**	**1,78**	**1,36**	Chemokine (C-X-C motif) ligand 2 (Cxcl2), mRNA	0.000000	9.900860	10.731600	5.500370	5.943020	1.005710	0.219786	0.360967	0.276840	CXCL2			
**Cxcl1**	**1,53**	**1,27**	Chemokine (C-X-C motif) ligand 1 (Cxcl1), mRNA	0.000000	9.728770	10.344500	6.920340	7.261270	0.513879	0.297036	0.143504	0.088698	CXCL2			
**Il1rn**	**1,57**	**1,04**	Interleukin 1 receptor antagonist (Il1rn), transcript variant 1, mRNA	0.000389	5.522350	6.175000	4.965300	5.026550	0.156723	0.249552	0.104468	0.097133	IL1RN			
**Cxcl10**	**1,95**	**0,93**	Chemokine (C-X-C motif) ligand 10, mRNA (cDNA clone MGC:41087 IMAGE:1446589)	0.000000	7.978030	8.942490	5.155360	5.057940	0.436640	0.214751	0.077327	0.060234	CXCL10			
**Il1r1**	**0,69**	**0,28**	Interleukin 1 receptor, type I (Il1r1), transcript variant 1, mRNA	0.000137	9.873190	9.327870	8.951560	7.095650	0.346828	0.252984	0.352454	0.118077	IL1R1			
**Extracellular matrix organization:**																
**Itgam**	**1,21**	**4,38**	Integrin alpha M (Itgam), transcript variant 2, mRNA	0.010774	7.750830	8.028900	6.112870	8.245180	0.722592	0.226547	0.504092	0.230229	ITGAM			
**Klk1b11**	**1,31**	**4,09**	Kallikrein 1-related peptidase b11, mRNA (cDNA clone MGC:19030 IMAGE:4167568)	0.000016	8.322660	8.709050	4.612760	6.646190	0.816289	0.443728	0.186354	0.376273	KLK3			

**Lum**	**1,11**	**3,54**	Lumican (Lum), mRNA	0.000000	10.024400	10.180800	6.316710	8.141040	0.466595	0.145198	0.297287	0.195125	LUM			
**Mfap5**	**1,12**	**3,46**	Microfibrillar associated protein 5, mRNA (cDNA clone MGC:35969 IMAGE:3982519)	0.000271	10.417500	10.576000	7.758620	9.550860	0.491401	0.237017	0.517309	0.197525	MFAP5			
**Ctss**	**0,99**	**3,44**	Cathepsin S, mRNA (cDNA clone MGC:6643 IMAGE:3495719)	0.007677	8.750960	8.743590	8.170280	9.953770	0.501668	0.168963	0.310807	0.203569	CTSS			
**Mmp9**	**0,89**	**3,43**	Matrix metallopeptidase 9 (Mmp9), mRNA	0.008268	8.616420	8.445340	6.563750	8.343030	0.912907	0.196311	0.289576	0.293522	MMP9			
**Itgb2**	**1,23**	**3,33**	Integrin beta 2 (Itgb2), mRNA	0.007205	8.052580	8.346560	6.607600	8.344590	0.595593	0.231850	0.375665	0.253054	ITGB2			
**Prss1**	**2,15**	**3,33**	Protease, serine, 1 (trypsin 1) (Prss1), mRNA	0.000010	11.769600	12.872100	11.552400	13.287100	0.293112	0.153288	0.146605	0.156251	PRSS3	FUMA mapped loci 500kb	1,36E-09	rs1758632
**Mmp12**	**1,62**	**3,29**	Matrix metallopeptidase 12, mRNA (cDNA clone MGC:29351 IMAGE:5037674)	0.001497	5.783990	6.479980	5.066180	6.785850	0.105178	0.288756	0.130283	0.485073	MMP12			
**Fbn1**	**1,08**	**3,13**	Fibrillin 1, mRNA (cDNA clone IMAGE:3483994)	0.000008	11.309800	11.418200	8.130820	9.775480	0.408001	0.167144	0.467295	0.214609	FBN1			
**Fn1**	**1,12**	**2,68**	Fibronectin 1, mRNA (cDNA clone IMAGE:4985138)	0.000072	11.659200	11.828700	9.298310	10.717900	0.390519	0.117131	0.414214	0.268105	FN1			
**Pcolce2**	**0,96**	**2,54**	Procollagen C-endopeptidase enhancer 2 (Pcolce2), mRNA	0.000016	9.386840	9.322630	6.586020	7.930860	0.408777	0.135187	0.404951	0.236958	PCOLCE2			
**Itga4**	**0,98**	**2,53**	Integrin alpha 4 (Itga4), mRNA	0.001012	7.824600	7.790080	6.435220	7.772460	0.373884	0.127596	0.218510	0.290798	ITGA4			
**Adamts5**	**0,93**	**2,43**	Putative secreted metalloprotease ADAMTS5 (Adamts5)	0.000018	10.007900	9.910090	7.080640	8.361570	0.503182	0.064537	0.430158	0.181404	ADAMTS5			
**Col14a1**	**1,04**	**2,37**	Collagen type XIV, partial	0.000000	10.672000	10.728500	7.853340	9.096750	0.283260	0.119400	0.280174	0.168760	COL14A1			
**Dcn**	**0,97**	**2,36**	Decorin (Dcn), mRNA	0.000051	12.918300	12.878800	11.102400	12.338900	0.073850	0.037320	0.362211	0.082744	DCN			
**Ctsb**	**1,09**	**2,34**	Cathepsin B, mRNA (cDNA clone MGC:6211 IMAGE:3500700)	0.000001	11.007200	11.132500	9.538990	10.764300	0.234100	0.090870	0.126098	0.155693	CTSB			
**Aspn**	**1,05**	**2,33**	Asporin, mRNA (cDNA clone MGC:41375 IMAGE:1365428)	0.000000	9.953260	10.020900	6.641440	7.861580	0.422817	0.127212	0.340327	0.264632	ASPN			
**Mmp2**	**1,02**	**2,32**	Matrix metallopeptidase 2 (Mmp2), mRNA	0.000000	11.032800	11.060200	7.417450	8.632090	0.430149	0.095528	0.231395	0.184571	MMP2			
**Col12a1**	**1,83**	**2,32**	Collagen, type XII, alpha 1 (Col12a1), mRNA	0.000219	6.877460	7.747120	5.501910	6.713370	0.422368	0.342439	0.138546	0.207915	COL12A1			
**Lama2**	**0,95**	**2,29**	Laminin, alpha 2, mRNA (cDNA clone IMAGE:5151000)	0.000000	10.323000	10.251800	7.189830	8.386530	0.404194	0.069292	0.251114	0.141798	LAMA2			
**Pcolce**	**1,09**	**2,15**	Procollagen C-endopeptidase enhancer protein (Pcolce), mRNA	0.000000	11.120200	11.240800	7.539330	8.641030	0.261017	0.081487	0.213401	0.143125	PCOLCE			
**Itga11**	**0,81**	**2,12**	Integrin alpha 11, mRNA (cDNA clone MGC:76390 IMAGE:6842988)	0.000016	7.979030	7.676910	5.563540	6.648720	0.515688	0.197709	0.149790	0.177139	ITGA11			
**Nid1**	**1,01**	**2,08**	Nidogen 1 (Nid1), mRNA	0.000000	11.412000	11.426200	8.347080	9.406850	0.340046	0.078905	0.321319	0.183608	NID1			
**Emilin2**	**1,07**	**1,99**	Strain C57BL/6J basilin	0.000094	8.939210	9.037210	6.773350	7.767020	0.508377	0.192330	0.307561	0.123770	EMILIN2			
**Lox**	**1,60**	**1,98**	Lysyl oxidase, mRNA (cDNA clone MGC:11525 IMAGE:2655752)	0.000000	9.429050	10.106100	5.390790	6.375120	0.446646	0.240528	0.335192	0.121649	LOX			
**Tgfb1**	**1,21**	**1,96**	Transforming growth factor, beta 1, mRNA (cDNA clone MGC:5747 IMAGE:3586216)	0.000979	8.323640	8.593110	7.209070	8.181330	0.369454	0.167550	0.166729	0.193095	TGFB1			
**Bmp4**	**0,98**	**1,94**	Bone morphogenetic protein 4, mRNA (cDNA clone MGC:31017 IMAGE:4192158)	0.000000	9.421920	9.393360	6.950590	7.903940	0.298413	0.101749	0.187984	0.225653	BMP4			
**Loxl1**	**1,24**	**1,89**	Lysyl oxidase-like 1, mRNA (cDNA clone MGC:46960 IMAGE:5251968)	0.000000	9.615280	9.926600	6.795440	7.714710	0.407435	0.174030	0.244443	0.152039	LOXL1			

**Ddr2**	**0,98**	**1,87**	Discoidin domain receptor family, member 2 (Ddr2), mRNA	0.000000	9.987380	9.957880	6.960970	7.863650	0.408476	0.079224	0.278239	0.204249	DDR2			
**Col8a1**	**1,47**	**1,79**	Collagen, type VIII, alpha 1, mRNA (cDNA clone MGC:18687 IMAGE:3674027)	0.000014	7.792170	8.347620	5.825070	6.664650	0.376244	0.333131	0.163358	0.108912	COL8A1			
**Col1a2**	**1,10**	**1,76**	Collagen, type I, alpha 2, mRNA (cDNA clone IMAGE:3481648)	0.000000	10.727200	10.868700	8.260330	9.076060	0.283138	0.084820	0.237623	0.109697	COL1A2			
**Vcan**	**1,31**	**1,74**	Versican (Vcan), transcript variant 2, mRNA	0.000277	8.841280	9.227050	6.324140	7.122160	0.596614	0.305581	0.458976	0.298704	VCAN			
**Col6a1**	**0,98**	**1,74**	Collagen, type VI, alpha 1, mRNA (cDNA clone IMAGE:3488924)	0.000000	10.209600	10.178000	7.657350	8.453450	0.285087	0.082631	0.176216	0.081540	COL6A1			
**Ppib**	**0,99**	**1,74**	Peptidylprolyl isomerase B, mRNA (cDNA clone MGC:6241 IMAGE:3483267)	0.000000	11.449000	11.436100	9.564720	10.360200	0.159771	0.044018	0.227152	0.139426	PPIB	FUMA mapped loci 500kb	2,58E-10	rs982077
**Col3a1**	**1,01**	**1,72**	Collagen, type III, alpha 1, mRNA (cDNA clone IMAGE:2812464)	0.000004	10.730400	10.749900	8.468140	9.247610	0.260853	0.095940	0.330072	0.157514	COL3A1			
**Ctsd**	**1,15**	**1,71**	Cathepsin D, mRNA (cDNA clone IMAGE:4500905)	0.000000	10.946200	11.148300	9.746980	10.518100	0.103423	0.071412	0.078343	0.142768	CTSD			
**Itgal**	**1,03**	**1,67**	Strain DBA/2J integrin alpha L (Itgal)	0.000543	6.167560	6.204550	5.204470	5.946420	0.196298	0.123570	0.140028	0.188707	ITGAL			
**Sdc3**	**1,02**	**1,62**	Syndecan 3 (Sdc3), mRNA	0.000014	8.239190	8.261700	6.862290	7.559000	0.207451	0.095111	0.136410	0.236119	SDC3			
**Itgax**	**1,07**	**1,61**	Integrin alpha X (Itgax), mRNA	0.007407	6.219190	6.316530	5.563930	6.252850	0.182495	0.112405	0.113610	0.260152	ITGAX			
**Htra1**	**0,89**	**1,61**	HtrA serine peptidase 1, mRNA (cDNA clone MGC:19188 IMAGE:4235912)	0.000000	9.776960	9.606690	6.151760	6.837000	0.244297	0.065925	0.244243	0.103425	HTRA1	FUMA mapped loci 500kb	1,48E-10	rs2421016
**Efemp1**	**0,93**	**1,59**	Epidermal growth factor-containing fibulin-like extracellular matrix protein 1 (Efemp1), mRNA	0.000000	11.812300	11.712200	8.683390	9.354720	0.155750	0.064631	0.272161	0.206861	EFEMP1			
**Fbln1**	**1,17**	**1,56**	Fibulin 1, mRNA (cDNA clone MGC:6128 IMAGE:3495754)	0.000000	10.388400	10.619600	7.264330	7.902600	0.321640	0.058344	0.241439	0.171913	FBLN1			
**Mmp13**	**0,95**	**1,54**	Matrix metallopeptidase 13 (Mmp13), mRNA	0.000013	6.470200	6.398140	4.642770	5.267040	0.465212	0.135678	0.065368	0.117710	MMP13			
**Ctsl**	**1,01**	**1,52**	Cell-line LXB2 cathepsin L	0.000000	11.141600	11.155100	8.852620	9.460050	0.097335	0.059216	0.132104	0.017877	CTSV			
**Col1a1**	**1,15**	**1,52**	Collagen, type I, alpha 1, mRNA (cDNA clone IMAGE:3586143)	0.000000	10.935000	11.135500	8.031760	8.631750	0.266243	0.121309	0.233688	0.149510	COL1A1			
**Lamb3**	**1,59**	**1,30**	Laminin, beta 3, mRNA (cDNA clone MGC:5994 IMAGE:3592888)	0.000033	6.810430	7.481970	5.984590	6.367420	0.205711	0.191636	0.131249	0.114386	LAMB3			
**Fbn2**	**1,71**	**1,22**	Fibrillin 2, mRNA (cDNA clone IMAGE:1498131)	0.000001	6.768030	7.538420	5.329110	5.620050	0.224668	0.299633	0.040245	0.055675	FBN2			
**Tnc**	**1,76**	**1,15**	Tenascin C (Tnc), mRNA	0.000441	5.826840	6.640920	4.638120	4.842080	0.255495	0.411747	0.209073	0.078331	TNC			
**Adamts4**	**1,81**	**1,00**	A disintegrin-like and metallopeptidase (reprolysin type) with thrombospondin type 1 motif, 4, mRNA (cDNA clone MGC:38401 IMA	0.000002	7.422570	8.274990	5.620910	5.623810	0.429219	0.329872	0.123510	0.054500	ADAMTS4			
**Sdc1**	**1,65**	**1,00**	Syndecan 1, mRNA (cDNA clone MGC:5879 IMAGE:3501359)	0.000000	7.570910	8.295550	6.103910	6.106430	0.239056	0.201607	0.129425	0.091373	SDC1			
**P4ha3**	**1,50**	**0,96**	Procollagen-proline, 2-oxoglutarate 4-dioxygenase (proline 4-hydroxylase), alpha polypeptide III, mRNA (cDNA clone MGC:99449	0.002296	5.797640	6.386700	5.547620	5.494480	0.111139	0.185985	0.154164	0.078522	P4HA3			
**Itgb4**	**1,59**	**0,88**	Integrin beta 4, mRNA (cDNA clone IMAGE:4911096)	0.000121	8.117690	8.786280	7.501780	7.324580	0.263819	0.191697	0.149422	0.125834	ITGB4			
**Mmp7**	**3,21**	**0,74**	Matrix metallopeptidase 7 (Mmp7), mRNA	0.000005	5.701670	7.384380	5.237300	4.804820	0.163177	0.212607	0.325335	0.017212	MMP7			

**Tgfb2**	**1,34**	**0,65**	Transforming growth factor, beta 2, mRNA (cDNA clone MGC:7998 IMAGE:3585774)	0.000000	8.188690	8.609740	6.511430	5.886580	0.212424	0.129726	0.208932	0.089005	TGFB2				
**Plec1**	**1,11**	**0,63**	Plectin 1, mRNA (cDNA clone IMAGE:30056202)	0.000068	7.896570	8.048640	7.566820	6.900610	0.126952	0.090782	0.157513	0.070855	PLEC				
**Jam2**	**0,93**	**0,63**	Junction adhesion molecule 2, mRNA (cDNA clone MGC:41518 IMAGE:1195543)	0.000001	9.357690	9.254660	8.224260	7.552090	0.105793	0.109259	0.207696	0.028363	JAM2				
**Itga1**	**0,96**	**0,61**	Pelota homolog (Drosophila) (Pelo), mRNA	0.001937	8.226910	8.172470	7.941020	7.221030	0.104735	0.121083	0.188407	0.133307	ITGA1				
**Adamts9**	**1,49**	**0,47**	MKIAA1312 protein	0.011971	7.085630	7.665710	7.668180	6.586250	0.255575	0.111564	0.250988	0.286620	ADAMTS9	GWAS mapped loci	FUMA mapped loci 500kb	1,63E-14	rs2292662; rs6795735
**Ddr1**	**1,13**	**0,58**	Discoidin domain receptor family, member 1 (Ddr1), transcript variant 1, mRNA	0.000000	8.953190	9.135040	7.724420	6.926620	0.224689	0.055470	0.158000	0.031879	DDR1				
**Tgfb3**	**1,30**	**0,57**	Transforming growth factor, beta 3, mRNA (cDNA clone MGC:18512 IMAGE:3663150)	0.000000	9.699090	10.081200	6.923770	6.114260	0.251386	0.107876	0.296974	0.086621	TGFB3				
**Actn1**	**1,27**	**0,56**	Actinin, alpha 1, mRNA (cDNA clone IMAGE:3483627)	0.000000	9.337390	9.677010	8.515980	7.688040	0.059878	0.095380	0.195348	0.117282	ACTN1				
**Sh3pxd2a**	**0,87**	**0,56**	SH3 and PX domains 2A (Sh3pxd2a), mRNA	0.000103	8.204820	8.007580	7.423950	6.586790	0.226939	0.105303	0.231464	0.055960	SH3PXD2A				
**Ncam1**	**1,15**	**0,56**	Neural cell adhesion molecule 1 (Ncam1), transcript variant 2, mRNA	0.000089	8.593590	8.795290	7.750490	6.912070	0.423095	0.159046	0.137418	0.173073	NCAM1				
**Lama5**	**1,61**	**0,54**	Laminin, alpha 5, mRNA (cDNA clone IMAGE:5037040)	0.000005	7.560770	8.246690	7.143920	6.260300	0.187215	0.098610	0.233039	0.037126	LAMA5				
**Lamc2**	**1,55**	**0,53**	Laminin gamma2 chain	0.000016	6.932980	7.566240	6.380720	5.476550	0.100474	0.159377	0.270348	0.088020	LAMC2				
**Dst**	**0,87**	**0,51**	Dystonin, mRNA (cDNA clone IMAGE:4950687)	0.000001	8.991930	8.792320	8.000640	7.037340	0.229912	0.093004	0.180658	0.079721	DST				
**Sdc4**	**1,19**	**0,51**	Syndecan 4, mRNA (cDNA clone MGC:11456 IMAGE:3154160)	0.000000	9.799060	10.046300	8.762300	7.783850	0.105296	0.053274	0.252806	0.087096	SDC4				
**Pecam1**	**1,09**	**0,50**	Platelet/endothelial cell adhesion molecule 1, mRNA (cDNA clone MGC:6104 IMAGE:3592726)	0.000013	9.253430	9.371790	8.581240	7.591960	0.157222	0.121905	0.228265	0.085967	PECAM1				
**Mmp15**	**1,70**	**0,48**	Matrix metallopeptidase 15 (Mmp15), mRNA	0.004336	7.734210	8.503300	7.778760	6.706650	0.180955	0.158793	0.406557	0.139451	MMP15				
**Kdr**	**0,96**	**0,44**	Kinase insert domain protein receptor, mRNA (cDNA clone MGC:18600 IMAGE:4238984)	0.000002	9.760660	9.702550	8.800720	7.620970	0.159694	0.127925	0.228985	0.155953	KDR				
**Tnr**	**0,98**	**0,42**	Tenascin R, mRNA (cDNA clone MGC:169667 IMAGE:8861062)	0.000284	7.720200	7.689320	6.642840	5.399800	0.581199	0.274774	0.156998	0.147198	TNR				
**Ttr**	**1,20**	**0,40**	Transthyretin, mRNA (cDNA clone MGC:18651 IMAGE:4192268)	0.000071	11.982600	12.246300	11.641100	10.324000	0.274580	0.107041	0.125602	0.380711	TTR				
**Spp1**	**1,91**	**0,40**	Osteopontin (OPN)	0.006955	10.534900	11.465600	10.326400	8.990050	0.126484	0.118604	0.612096	0.382895	SPP1				
**Nrxn1**	**0,95**	**0,37**	Neurexin I, mRNA (cDNA clone IMAGE:30544573)	0.001547	7.803720	7.727060	7.353230	5.925260	0.521531	0.247105	0.169862	0.160994	NRXN1				
**Agrn**	**1,52**	**0,36**	Agrin, mRNA (cDNA clone IMAGE:5006620)	0.000003	8.265700	8.872050	7.543700	6.086230	0.189956	0.124694	0.321784	0.156158	AGRN				
**F11r**	**1,36**	**0,34**	F11 receptor, mRNA (cDNA clone MGC:18960 IMAGE:3985305)	0.000000	9.184390	9.632210	8.197620	6.625440	0.200953	0.053423	0.301300	0.077528	F11R				
**Mme**	**1,36**	**0,34**	Membrane metallo endopeptidase, mRNA (cDNA clone MGC:31469 IMAGE:4483558)	0.042392	6.233220	6.671630	5.997090	4.420470	0.129938	0.212155	0.781174	0.059760	MME				
**Itga3**	**1,26**	**0,33**	Integrin alpha 3, mRNA (cDNA clone IMAGE:3991894)	0.000038	8.279990	8.616230	7.811010	6.229230	0.262482	0.102443	0.335037	0.152871	ITGA3				

**Mmp19**	**1,05**	**1,51**	Matrix metalloproteinase 19	0.000007	8.268550	8.337860	5.504480	6.101720	0.527538	0.181633	0.359960	0.101378	MMP19
**Mmp23**	**0,93**	**1,50**	Matrix metallopeptidase 23 (Mmp23), mRNA	0.000000	8.892500	8.795310	5.995290	6.582730	0.295929	0.094702	0.122223	0.111536	MMP23B
